# Genetic Variations of *PIP4K2A* Confer Vulnerability to Poor Antipsychotic Response in Severely Ill Schizophrenia Patients

**DOI:** 10.1371/journal.pone.0102556

**Published:** 2014-07-15

**Authors:** Harpreet Kaur, Ajay Jajodia, Sandeep Grover, Ruchi Baghel, Meenal Gupta, Sanjeev Jain, Ritushree Kukreti

**Affiliations:** 1 Genomics and Molecular Medicine, CSIR- Institute of Genomics and Integrative Biology, Delhi, India; 2 Department of Psychiatry, National Institute of Mental Health and Neuro Sciences, Bengaluru, Karnataka, India; Tel Aviv University, Israel, Israel

## Abstract

Literature suggests that disease severity and neurotransmitter signaling pathway genes can accurately identify antipsychotic response in schizophrenia patients. However, putative role of signaling molecules has not been tested in schizophrenia patients based on severity of illness, despite its biological plausibility. In the present study we investigated the possible association of polymorphisms from five candidate genes *RGS4*, *SLC6A3*, *PIP4K2A*, *BDNF*, *PI4KA* with response to antipsychotic in variably ill schizophrenia patients. Thus in present study, a total 53 SNPs on the basis of previous reports and functional grounds were examined for their association with antipsychotic response in 423 schizophrenia patients segregated into low and high severity groups. Additionally, haplotype, diplotype, multivariate logistic regression and multifactor-dimensionality reduction (MDR) analyses were performed. Furthermore, observed associations were investigated in atypical monotherapy (n = 355) and risperidone (n = 260) treated subgroups. All associations were estimated as odds ratio (OR) and 95% confidence interval (CI) and test for multiple corrections was applied. Single locus analysis showed significant association of nine variants from *SLC6A3*, *PIP4K2A* and *BDNF* genes with incomplete antipsychotic response in schizophrenia patients with high severity. We identified significant association of six marker diplotype ATTGCT/ATTGCT (rs746203-rs10828317-rs7094131-rs2296624-rs11013052-rs1409396) of *PIP4K2A* gene in incomplete responders (corrected p-value = 0.001; adjusted-OR = 3.19, 95%-CI = 1.46–6.98) with high severity. These associations were further observed in atypical monotherapy and risperidone sub-groups. MDR approach identified gene-gene interaction among *BDNF*_rs7103411-*BDNF*_rs1491851-*SLC6A3*_rs40184 in severely ill incomplete responders (OR = 7.91, 95%-CI = 4.08–15.36). While *RGS4*_rs2842026-*SLC6A3*_rs2975226 interacted synergistically in incomplete responders with low severity (OR = 4.09, 95%-CI = 2.09–8.02). Our findings provide strong evidence that diplotype ATTGCT/ATTGCT of *PIP4K2A* gene conferred approximately three-times higher incomplete responsiveness towards antipsychotics in severely ill patients. These results are consistent with the known role of phosphatidyl-inositol-signaling elements in antipsychotic action and outcome. Findings have implication for future molecular genetic studies as well as personalized medicine. However more work is warranted to elucidate underlying causal biological pathway.

## Introduction

Schizophrenia is a heterogeneous mental illness with varying degrees of positive, negative and cognitive symptoms governed by both genetic and environmental factors. Accumulating literature suggests contribution of genetic variations may lead to differential antipsychotic response ranging from adequate response, to development of side-effects or treatment resistance [1]. Additionally affected individuals may have different combinations of disease symptoms which cause considerable disease heterogeneity and differential therapeutic outcome [2]. Polygenic involvement of genetic factors in the schizophrenia pathophysiology hinders the identification and characterization of causal genes that could develop as therapeutic targets. Inspite of the increasing number of pharmacogenomic studies attempts to explicitly uncover the mechanism behind the antipsychotic response remains inadequate which urges further investigation of candidate pathway genes including drug targets and signaling molecules since effect of antipsychotic drugs propagated through various biochemical processes that are part of coherent signaling network. Thus, it is important to investigate the influence of genetic variation and clinical factors on therapeutic outcome in differentially affected schizophrenia individuals according to the disease severity [3]. These facts prompted us to identify variants that may influence drug response by investigating critical signaling molecules known to express in different brain regions, implicated in schizophrenia and drug response. Among these, we prioritized Brain-derived neurotrophic factor (*BDNF*), Solute carrier family 6 (neurotransmitter transporter), member 3 (*SLC6A3*), Regulator of G-protein signaling 4 (*RGS4*), Phosphatidylinositol-5-phosphate 4-kinase, type II, alpha (*PIP4K2A*) and Phosphatidylinositol 4-kinase, catalytic, alpha (*PI4KA*).

The *BDNF* is known to influence neurogenesis, neuroplasticity and interact with other neurotransmitters like dopamine, glutamate, serotonin and gamma-aminobutyric acid. Polymorphisms in this gene have been studied for roles in efficacy and adverse effects of antipsychotics treatment [4]. Recently it is suggested that antidepressant and antipsychotic treatments ameliorates disturbed monoamine systems caused by reduced *BDNF* activity, while reduced *BDNF* levels lead to differential treatment response in schizophrenia patients caused by stress and lack of neuronal activity [5]. *SLC6A3* plays a critical role in controlling dopamine transmission (spatial and temporal domains) through the accumulation of dopamine in extracellular space which is major site of action of psychostimulant drugs [6]–[8]. Sjoholm *et al*., reported increased number of *SLC6A3* binding sites in the schizophrenic patients who were being treated with dopamine D(2)-receptor blocking antipsychotics. Thus altered *SLC6A3* functioning may lead to inter-individual variability towards antipsychotic drug treatment [9]. Whereas reduced RGS expression may result in prolonged signal transduction, thereby leading to altered neurotransmission of dopamine, serotonin or glutamate. In addition, studies showed *RGS4* is extremely responsive to external stimuli and hence can modulate GPCR neurotransmitter receptors by antipsychotics [10], [11]. *PIP4K2A* and *PI4KA* have been suggested as putative susceptibility genes on both positional and functional grounds as these are part of phosphoinositide signaling pathway which is implicated in schizophrenia etiology and may modulate antipsychotic response [12]–[16]. *PI4KA* located at 22q11.2 deletion region repeatedly linked to schizophrenia other psychiatric diseases. While *PIP5K2A* maps to 10p12 region that has been implicated in schizophrenia and bipolar disorder. These two kinases involved in the biosynthesis of phosphatidylinositol-4,5-bisphosphate (PIP2) by phosphorylation of inositol ring at D4 position. PIP2, secondary messenger, is one of the key elements of crossroads in phosphoinositide signaling and plays a role in membrane transduction of neurotransmitter signals as well as in intracellular signaling pathways that are known to be implicated in schizophrenia [17].

Studies across populations could help to identify true genetic association but population structure and divergent linkage disequilibrium (LD) pattern have impact on associations which has lead to inconsistent results. Also, selection of markers under investigation could sufficiently contribute to non-replicability of associations. In addition to this, single marker expose only the localized effect if individual SNP being analyzed may not be the causal variant or in LD with the functional variant [18], [19]. Thus haplotype (multimarker) analyses may overcome differences among LD structure across populations and could be more informative in terms of identification of causal marker for disease development and therapeutic outcome [20]–[24].

With this background, in the present study, we investigated 53 polymorphisms from five genes (*RGS4*, *SLC6A3*, *PIP4K2A*, *BDNF* and *PI4KA*) in 423 south Indian schizophrenia cases segregated into low and high severity of illness to assess their influence on antipsychotic response. We observed association of six marker diplotype ATTGCT/ATTGCT (rs746203-rs10828317-rs7094131-rs2296624-rs11013052-rs1409396) of *PIP4K2A* gene with an estimated odds ratio of OR = 3.19 (95%-CI = 1.46–6.98) in severely ill schizophrenia patients who had inadequate response for antipsychotics irrespective of treatment in the study population. This further suggests that endophenotypically distinct subgroups of schizophrenia patients should considered separately for personalized therapeutic approaches.

## Methods

### Participant recruitment and clinical assessment

Our study is a pharmacogenetic based observational follow-up study conducted in a natural setting. The study sample comprised of 482 unrelated schizophrenia patients of south Indian origin, who met DSM-IV criteria for schizophrenia diagnosis (Table 1). These patients were recruited from the clinical services of NIMHANS during the period of March 2007 to March 2010. Best-estimate diagnosis was established on the basis of structured interviews by experienced psychiatrists using Schedules for Clinical Assessment in Neuropsychiatry (SCAN), OPCRIT 3.1 [25], [26]. Patients were excluded if they had 1) past history or family history of any mental illness or neurological disorder, 2) history of substance dependence or abuse, 3) history of head injury or 4) pregnancy. Detailed clinical and treatment history including age of onset (AOO), duration of illness (DOI), medication, relapse, non-compliance and change of treatment were also documented. Further, patients were evaluated by clinical global impressions (CGI) scale at the time of enrolment and each component of the CGI was rated separately to assess the patient’s status. This was followed by three months during which patients were treated with antipsychotic drugs. Medication was as chosen by the clinicians and administered drugs included typical antipsychotics such as chlorpromazine (200–400 mg/day), fluphenazine (20–50 mg/day), and flupenthixol (20–40 mg/day), and atypical antipsychotics such as clozapine (25–400 mg/day), risperidone (2–10 mg/day), olanzapine (5–20 mg/day), ziprasidone (40–160 mg/day), quetiapine (1200 mg/day), aripiprazole (10–45 mg/day), amisulpride (100–600 mg/day), and levosulpride (200 mg/day) or combination of two or more atypical/typical antipsychotic drugs. After three months of treatment, patients were invited again and re-evaluated with CGI for antipsychotic response. Total 423 patients completed three months follow-up period, among whom 355 (83.92%) were on atypical monotherapy, and 260 (61.45%) were on risperidone (Table 2). It is expected that severely ill patients would have poor treatment outcome, especially if they do not respond to several months of treatment or if they are not responding, their symptoms may become more severe. Taking this fact into consideration, patients with CGI-Severity (CGI-S) score ≤3 (not at all ill to mildly ill) were categorized into low severity group (LSG) and those with CGI-S ≥4 (moderately to most extremely ill) were categorized into high severity group (HSG) [3]. Further, patients with CGI-Improvement (CGI-I) ≤2 or a drop of 2 from baseline score were described as complete responders (CR) towards antipsychotic treatment. Patients with CGI-I ≥3 or more were grouped as incomplete responders (IR) [27]. Based on these criteria, 193 (45.63%) patients were grouped into LSG, among them 142 (33.57%) were CR and 51(12.06%) were IR and, 230 (54.37%) constituted HSG group with 68 (16.07%) CR and 162 (38.30%) IR. Additionally 230 healthy individuals without any history of psychiatric illness were recruited from the same geographical area. Ethnicity of participants was assessed by self-report.

**Table 1:** Demographic and clinical characteristics of schizophrenia cases (n = 482) and healthy controls (n = 230) enrolled in the study.

Characteristics(mean±SD)	Cases(n = 482)	Males (n = 289; 60%)	Female (n = 193; 40%)	aP-value
Age (yrs)	29.52±7.33	29.18±6.82	30.06±8.05	0.39
Age at onset (yrs)	24.98±7.11	24.73±6.41	25.36±8.06	0.96
Duration of Illness (yrs)	4.33±3.25	4.33±3.52	4.35±2.78	0.51
Disease Severity (CGI-S scale)	3.53±1.43	3.59±1.41	3.43±1.48	0.23
	**Controls (n = 230)**	**Males (n = 142; 62%)**	**Females (n = 86; 38%)**	a **P-value**
Age (mean±SD) yrs	29.48±8.51	30.64±7.31	27.61±9.01	0.01

**Footnote:** SD, Standard deviation.

a P-values were calculated by using two-tailed Student’s *t*-test.

**Table 2:** Demographic and clinical features of schizophrenia patients who had completed 3-months follow-up (n = 423).

Groups^a^ Parameters		Patients with low severity (LSG) (n = 193; 45.6%)				Patients with high severity (HSG) (n = 230; 54.4%)			
		CR n (%)	IR n (%)	OR (95% CI)	P-value^d^	CR n (%)	IR n (%)	OR (95%CI)	P-value^d^
Gender	Male	80 (56.4)	30 (58.8)	0.9 (0.45–1.81)	0.76	40 (58.8)	103 (63.6)	0.82 (0.44–1.53)	0.5
	Female	62 (44.6)	21 (41.2)			28 (41.2)	59 (36.4)		
Age (mean±SD) yrs		29.9±7.7	28.8±7.7	–	0.38^e^	28.68±6.1	29.39±7.1	–	0.48^e^
Age at onset^b^	Early	84 (59.1)	30 (60.0)	0.97 (0.47–1.95)	0.92	41 (60.3)	100 (61.7)	0.94 (0.51–1.76)	0.84
	Late	58 (40.9)	20 (40.0)			27 (39.7)	62 (38.3)		
Duration of illness^c^	Short	95 (67.9)	29 (59.2)	1.45 (0.70–3.0)	0.27	46 (70.8)	86 (53.4)	2.11 (1.09–4.15)	0.02
	Long	45 (32.1)	20 (40.8)			19 (29.2)	75 (46.6)		
Drug	typical+multi	19 (13.0)	5 (9.8)	1.42 (0.47–5.14)	0.51	10 (14.7)	34 (20.4)	0.64 (0.26–1.46)	0.26
	atypical	123 (87.0)	46 (90.2)			58(85.3)	128 (79.6)		

**Footnote:** LSG, Low Severity Group; HSG, High Severity Group; CR, Complete Responders; IR, Incomplete Responders; SD, Standard deviation; OR, Odds ratio; CI, Confidence interval.

a Low severity ≤3 CGI-S and High severity ≥4 CGI-S.

b early onset <25 yrs; late onset ≥25 yrs,

c short duration <4 yrs; long duration ≥4yrs.

d P-values were calculated by using Pearson’s χ^2^-test and^ e^P-values were calculated by using two-tailed Student’s *t*-test.

### Ethics Statement

Written informed consent was obtained from all the participants after clinicians explained the study protocol to them. The study was approved by institutional review board of both the participating institutes: CSIR-Institute of genomics and integrative biology (CSIR-IGIB) and National Institute of Mental Health and Neuro Sciences (NIMHANS). Informed consent was obtained from respective family members if patient was not judged to be take his or her own decisions or think appropriately. This assessment of clinical judgment was performed by primary care physicians and senior psychiatric clinicians.

### Population stratification

A major concern of association studies was genetic heterogeneity which may lead to false positive results [28]. Due to this reason, study participants were recruited from the same geographic region and were also genotyped for 10 autosomal microsatellite markers unlinked to schizophrenia for genetic homogeneity testing (Applied Biosystems linkage mapping set, version 2.0). These microsatellite markers were analyzed using GeneScan module of the Genotyper software, version 3.7 (Applied Biosystems). Stratification was tested by comparing genotype frequencies of each of the marker between patient and control groups using Pearson’s χ^2^ tests. The observed test statistics p-value was estimated as the fraction of 10,000 simulated test statistics which exceeded the observed value. Further, for each locus, the sum of test statistics was computed with the number of degrees of freedom (df) which was equal to the sum of the number of df for the individual loci. This was performed by Structured Population Association Test (STRAT) program (Table S1a in File S1) [29]. In addition to unlinked microsatellite markers, we selected 441 neutral markers to test presence of substructures in the studied population (Table S1b in File S1). These markers were selected from 4991 neutral markers from Affymetrix 50K dataset [30], [31]. Neutral markers which are not located in region linked to psychiatric illness and in genes known to be implicated in brain diseases, brain development and functioning were selected for present study. To test population stratification if any, the STRUCTURE software was used and admixture model was assumed [32]. According to this model individual *i* have inherited some fraction of his/her genome from ancestors in population *K* and it computes the proportion of the genome of an individual originating from each inferred population posterior mean estimates known as *Q*. This model is recommended due to its flexibility for dealing with real population complexities. We assumed *K* = 2 and model parameters includes length of burning period, number of Markov Chain Monte Carlo (MCMC) reps after burning, and iterations as 100, 100, and 100 respectively were considered.

### Selection of SNPs

Most obvious candidates for pharmacogenetic studies are the genes that encode the drug targets. However, genes that might modulate drug–target interactions and downstream signaling cascade are also important [33]. Therefore, genes that are involved in neurotransmitter signaling and are known to modulate antipsychotic response namely *RGS4*, *SLC6A3*, *PIP4K2A*, *BDNF* and *PI4KA* were selected for present study. All the investigated polymorphisms were either previously reported and or were observed as functional in repositories such as, RegulomeDB and HaploReg that were used to screen the possible functional and regulatory effect of variants on gene regulation [34], [35]. Non-coding SNPs located upstream and downstream of the gene spanning regulatory regions, predicted to be present in splice site, and enhancer or silencer elements and untranslated region and coding SNPs (synonymous and non-synonymous) were selected to cover the entire gene. Total 117 SNPs were prioritized across five selected genes, of which 113 SNPs passed assay designing optimization and hence genotyped in entire sample set (Table S2 in File S1).

### Genotyping

Peripheral whole blood samples were obtained from all the participants by venepuncture, and genomic DNA was prepared using a modified salting-out procedure [36]. Prioritized SNPs were genotyped by primer extension reaction followed by the MALDI-TOF mass spectrometry (Sequenom). Automated calls were made using Mass Array Typer module of the software. To rule out any genotyping error, 5% of the total samples were re-genotyped randomly using single-base primer extension methods (SNaPshot, Applied Biosystems). Calls were made after analyzing data in PeakScanner Software (Applied Biosystems). Allele calls, clustering patterns and peak intensities were reviewed by an experienced technician. The SNPs with call rate exceeding 98% were considered.

### Statistical analyses

Our analyses were focused on comparing the response of antipsychotics in presence of polymorphisms and adjusted for variables including gender, age, AOO, DOI, treatment type.

#### Genotype-Phenotype association analyses

Genotyping was completed on a total of 113 SNPs; 12 SNPs had missing genotype data for all participants. The remaining 101, were tested for HWE using χ^2^-test at 0.001 level of significance and allelic frequencies of all the variants were calculated for control and patient sample pools using statistical modules implemented in PLINK software [37]. Eight SNPs failed the HWE test (P<0.001) in controls and 17 SNPs failed in the minor allele frequency (MAF<0.1) test. Linkage disequilibrium (LD) among SNPs was estimated by correlation coefficient (r^2^) values for genotype data of all markers across the selected genes in controls using Tagger algorithm in Haploview program, version 4.1. [38]. Further, 23 SNPs were pruned out based on LD (r^2^>0.9) with other genotyped SNPs in the studied population and 53 SNPs were included in analyses (Table S2 and S3 in File S1).

All genotype-phenotype association analyses were performed on 53 SNPs. In order to reduce number of statistical tests, dominant and recessive model of inheritance were selected to compare their distribution in LSG and HSG for treatment response in 423 patients. These genetic models of inheritance were selected on the basis of distribution of genotypes and alleles of each SNP. To assess the study-wise significance, 10,000 permutation test were performed to obtained empirical p-values for all SNPs with the aim of calculating a value that controls for all the applied tests. Power of the study was calculated by PS Power and Sample Size Calculations, version 3.0, considering most significant p-value in single marker genotype-phenotype association analysis [39]. Entire genotype data was phased before constructing the haplotypes using the software PHASE 2.1 based on Bayesian algorithm. Parameter values of 100 iterations, a thinning interval of 10, and a burn-in value of 100 in the MCMC simulations were used [40]. SNPs found to be associated with drug response were included for haplotype analysis, using PLINK software and highly conservative Bonferroni method of multiple test correction was applied (p = 0.0027 = 0.05/18 p<0.05/n, where n = total number of statistical tests). Haplotypes that remained significant after correction were further examined for diplotype (homozygous condition). Additionally, p-values of single marker and diplotype were adjusted for gender, age, AOO, and DOI using multivariate logistic regression (STATA 11.0).

#### Subgroup analysis

The SNP, haplotype and diplotype that were observed to be associated with drug response in entire schizophrenia samples were also examined separately in subgroups with 355 patients on atypical antipsychotic monotherapy and out of these 260 patients were on risperidone.

#### Epistasis analyses

Considering the heterogeneous antipsychotic response in schizophrenia patients one can expect interaction among the SNPs. Therefore, all the 53 SNPs were evaluated for potential interactions using multifactor dimensionality reduction (MDR) method [41]. In brief, MDR is a non-parametric method that defines high and low-risk groups by combining lower and higher predisposing genotypes, which are then evaluated for its ability to categorize and predict drug response status through 10-fold cross validation. The cross validation consistency (CVC) was calculated for each possible model. Dendrogram were applied to portray SNPs interactions of the best model.

## Results

### Demographic and clinical characteristics of participants

Out of 482 recruited schizophrenia patients 423 subjects (87.8%) had completed three months of follow up (male:253; 59.8%, female:170; 40.2%). The majority of patients were diagnosed with paranoid schizophrenia (Table 1). The demographic characteristics of 423 schizophrenia patients eligible for genotype-phenotype correlation are summarized in Table 2. There was no significant gender difference among CR and IR in both the severity groups. In addition, no significant differences were observed in the age, AOO and treatment type among all the phenotypic groups studied except for DOI among CR and IR patients with high severity (unadjusted p-value −0.02). Additionally, treatment type was same in both severity groups.

Population stratification results of ten unlinked microsatellite markers suggested homogeneity among analyzed sample sets with p-value of 0.142 (χ^2^ = 109.79; df = 95) (Table S1a in File S1). Further, STRUCTURE analysis of 441 neutral markers revealed both cases and controls have same probability to be member of *K* = 2 clusters. Each individual in the data set represented by single vertical line divided into 2 colored segments (*K* = 2) that represents estimated membership fraction of each individual in both the *K* inferred clusters. Proportion of colored segments shows estimates of *Q* which is posterior mean estimate of each individual originating from each inferred population (Figure S1). In the summary plot depicts no clear distinction between the two assumed clusters hence we can conclude that studied samples were taken from same population pool. These results were further validated using STRAT software which computes summation of χ^2^ **= **353.603 (434 df), Global P-value after 1000 simulations for each locus found to be 0.9981 further supports that our studied sample pool is homogenous (Table S1b in File S1).

### Drug response analyses

In 423 patients, 9 SNPs showed statistical significant difference among CR and IR in LSG or HSG with p-value <0.05 based on the best model fit (dominant or recessive model) (Table 3). Furthermore, p-values were adjusted for age, gender, DOI, AOO and drugs. Polymorphism rs1409396 (*PIP4K2A*) showed most significant association (corrected p-value = 0.02, adjusted OR = 0.32, 95%-CI = 0.16–0.64) among complete and incomplete responders in HSG and remained significant after correcting for multiple comparisons using 10,000 permutations. Hence, patients in HSG having TT genotype are 68% less likely to respond towards antipsychotic drugs. The power of association was 0.963 with studied sample size, true OR for drug response in severely ill IR relative to CR was 0.32 and the associated with this test of this null hypothesis was 0.05. Same associations were detected in 355 atypical monotherapy and 260 risperidone sub-group. As expected, the influence of particular drug was not observed because *PIP4K2A* is element of neurotransmitter signaling pathway and involved in both schizophrenia manifestation and downstream signaling cascade activated after drug-receptor interactions.

**Table 3:** Association analyses of variants with antipsychotic response among schizophrenia patients with low and high severity of illness.

**All patients (n = 423)**									
**rsID**	**Test**	**Low severity group(n = 193)**				**High severity group(n = 230)**			
		**Complete responders**	**Incomplete responders**	**OR** a **(95%CI)**	**P-value** a	**Complete responders**	**Incomplete responders**	**OR** a **(95%CI)**	**P-value** a
**SLC6A3-5p15.3**									
rs2550956	Dom	TT+CT(6+56)/CC(80)	TT+CT(7+20)/CC(24)	1.49(0.76–2.91)	0.245	TT+CT(7+22)/CC(39)	TT+CT(17+72)/CC(73)	1.53(0.84–2.77)	0.161
	Rec	TT(6)/CC+CT(80+56)	TT(7)/CC+CT(24+20)	4.29(1.31–14.02)	0.016	TT(7)/CC+CT(39+22)	TT(17)/CC+CT(73+72)	0.9(0.33–2.5)	0.845
rs6347	Dom	GG+AG(5+31)/AA(106)	GG+AG(2+12)/AA(37)	1.2(0.57–2.54)	0.627	TT+GT(0+3)/GG(65)	TT+GT(0+12)/GG(150)	0.43(0.22–0.81)	**0.010**
	Rec	GG(5)/AA+AG(106+31)	GG(2)/AA+AG(37+12)	0.96(0.17–5.37)	0.962	GG(4)/AA+AG(43+21)	GG(0)/AA+AG(127+35)	0(0–0)	0
**PIP5K2A-10p12.2**									
rs10828317	Dom	CC+CT(4+39)/TT(99)	CC+CT(1+18)/TT(32)	1.74(0.84–3.58)	0.136	CC+CT(2+29)/TT(37)	CC+CT(3+49)/TT(110)	0.5(0.27–0.93)	**0.028**
	Rec	CC(4)/TT+CT(99+39)	CC(1)/TT+CT(32+18)	1.24(0.12–12.87)	0.855	CC(2)/TT+CT(37+29)	CC(3)/TT+CT(110+49)	0.49(0.08–3.05)	0.441
rs11013052	Dom	AA+AC(8+39)/CC(95)	AA+AC(1+20)/CC(30)	1.63(0.81–3.27)	0.170	AA+AC(4+28)/CC(36)	AA+AC(2+53)/CC(107)	0.51(0.28–0.95)	**0.032**
	Rec	AA(8)/CC+AC(95+39)	AA(1)/CC+AC(30+20)	0.4(0.05–3.34)	0.394	AA(4)/CC+AC(36+28)	AA(2)/CC+AC(107+53)	0.18(0.03–1.04)	0.055
rs1409396	Dom	CC+CT(19+70)/TT(53)	CC+CT(6+25)/TT(20)	1.03(0.51–2.07)	0.938	CC+CT(14+41)/TT(13)	CC+CT(15+77)/TT(70)	0.34(0.17–0.68)	**0.002** b
	Rec	CC(19)/TT+CT(53+70)	CC(6)/TT+CT(20+25)	0.91(0.33–2.48)	0.849	CC(14)/TT+CT(13+41)	CC(15)/TT+CT(70+77)	0.36(0.16–0.82)	**0.015**
rs2296624	Dom	AA+AG(5+38)/GG(99)	AA+AG(1+18)/GG(32)	1.79(0.87–3.69)	0.117	AA+AG(2+29)/GG(37)	AA+AG(3+49)/GG(110)	0.51(0.28–0.94)	**0.032**
	Rec	AA(5)/GG+AG(99+38)	AA(1)/GG+AG(32+18)	0.97(0.1–9.35)	0.980	AA(2)/GG+AG(37+29)	AA(3)/GG+AG(110+49)	0.56(0.09–3.51)	0.534
rs7094131	Dom	CC+CT(5+48)/TT(89)	CC+CT(1+19)/TT(31)	1.28(0.64–2.57)	0.485	CC+CT(2+35)/TT(31)	CC+CT(3+57)/TT(102)	0.42(0.23–0.78)	**0.006**
	Rec	CC(5)/TT+CT(89+48)	CC(1)/TT+CT(31+19)	0.86(0.09–8.13)	0.895	CC(2)/TT+CT(31+35)	CC(3)/TT+CT(102+57)	0.56(0.09–3.52)	0.537
rs746203	Dom	GG+AG(9+60)/AA(73)	GG+AG(1+21)/AA(29)	0.88(0.45–1.73)	0.717	GG+AG(7+32)/AA(29)	GG+AG(6+63)/AA(93)	0.47(0.26–0.86)	**0.015**
	Rec	GG(9)/AA+AG(73+60)	GG(1)/AA+AG(29+21)	0.33(0.04–2.76)	0.305	GG(7)/AA+AG(29+32)	GG(6)/AA+AG(93+63)	0.27(0.08–0.87)	**0.028**
**BDNF-11p13**									
rs56164415	Dom	AA+AG(9+52)/GG(81)	AA+AG(3+13)/GG(35)	0.59(0.29–1.2)	0.147	AA+AG(3+12)/GG(53)	AA+AG(13+54)/GG(95)	2.63(1.31–5.29)	**0.007**
	Rec	AA(9)/GG+AG(81+52)	AA(3)/GG+AG(35+13)	0.87(0.22–3.52)	0.846	AA(3)/GG+AG(53+12)	AA(13)/GG+AG(95+54)	1.85(0.49–6.94)	0.361
**Atypical monotherapy group (n = 355)**									
**rsID**	**Test**	**Low severity group (169)**				**High severity group (n = 186)**			
		**Complete responders**	**Incomplete responders**	**OR (95%CI)**	**P-value**	**Complete responders**	**Incomplete responders**	**OR (95%CI)**	**P-value**
**SLC6A3-5p15.3**									
rs2550956	Dom	TT+CT(6+50)/CC(67)	TT+CT(7+18)/CC(21)	1.47(0.72–2.99)	0.288	TT+CT(6+19)/CC(33)	TT+CT(13+60)/CC(55)	1.53(0.84–2.77)	0.161
	Rec	TT(6)/CC+CT(67+50)	TT(7)/CC+CT(21+18)	4.34(1.32–14.28)	0.016	TT(6)/CC+CT(33+19)	TT(13)/CC+CT(55+60)	0.85(0.27–2.67)	0.782
rs6347	Dom	GG+AG(5+27)/AA(91)	GG+AG(2+10)/AA(34)	1.09(0.49–2.44)	0.830	GG+AG(4+19)/AA(35)	GG+AG(0+26)/AA(102)	0.43(0.22–0.81)	**0.010**
	Rec	GG(5)/AA+AG(91+27)	GG(2)/AA+AG(34+10)	0.97(0.17–5.55)	0.969	GG(4)/AA+AG(35+19)	GG(0)/AA+AG(102+26)	0(0–0)	0
**PIP5K2A-10p12.2**									
rs10828317	Dom	CC+CT(4+34)/TT(85)	CC+CT(1+16)/TT(29)	1.73(0.8–3.76)	0.167	CC+CT(2+24)/TT(32)	CC+CT(2+35)/TT(91)	0.5(0.27–0.93)	**0.028**
	Rec	CC(4)/TT+CT(85+34)	CC(1)/TT+CT(29+16)	1.38(0.13–14.51)	0.790	CC(2)/TT+CT(32+24)	CC(2)/TT+CT(91+35)	0.28(0.04–2.17)	0.224
rs11013052	Dom	AA+AC(7+34)/CC(82)	AA+AC(1+18)/CC(27)	1.61(0.77–3.38)	0.209	AA+AC(4+23)/CC(31)	AA+AC(1+36)/CC(91)	0.51(0.28–0.95)	**0.032**
	Rec	AA(7)/CC+AC(82+34)	AA(1)/CC+AC(27+18)	0.42(0.05–3.66)	0.436	AA(4)/CC+AC(31+23)	AA(1)/CC+AC(91+36)	0.07(0.01–0.72)	**0.025**
rs1409396	Dom	CC+CT(17+61)/TT(45)	CC+CT(6+23)/TT(17)	1.13(0.54–2.39)	0.747	CC+CT(13+34)/TT(11)	CC+CT(10+61)/TT(57)	0.34(0.17–0.68)	**0.002**
	Rec	CC(17)/TT+CT(45+61)	CC(6)/TT+CT(17+23)	1.02(0.36–2.85)	0.973	CC(13)/TT+CT(11+34)	CC(10)/TT+CT(57+61)	0.22(0.09–0.58)	**0.002**
rs2296624	Dom	AA+AG(3+36)/GG(84)	AA+AG(1+12)/GG(33)	1.84(0.84–4.01)	0.127	AA+AG(2+24)/GG(32)	AA+AG(1+35)/GG(92)	0.51(0.28–0.94)	**0.032**
	Rec	AA(3)/GG+AG(84+36)	AA(1)/GG+AG(33+12)	1.05(0.1–10.61)	0.965	AA(2)/GG+AG(32+24)	AA(1)/GG+AG(92+35)	0.15(0.01–1.77)	0.131
rs7094131	Dom	CC+CT(5+40)/TT(78)	CC+CT(1+17)/TT(28)	1.31(0.63–2.75)	0.468	CC+CT(2+30)/TT(26)	CC+CT(1+44)/TT(83)	0.42(0.23–0.78)	**0.006**
	Rec	CC(5)/TT+CT(78+40)	CC(1)/TT+CT(28+17)	0.91(0.1–8.75)	0.938	CC(2)/TT+CT(26+30)	CC(1)/TT+CT(83+44)	0.15(0.01–1.86)	0.141
rs746203	Dom	GG+AG(8+52)/AA(63)	GG+AG(1+19)/AA(26)	0.9(0.44–1.83)	0.765	GG+AG(6+27)/AA(25)	GG+AG(3+51)/AA(74)	0.47(0.26–0.86)	**0.015**
	Rec	GG(8)/AA+AG(63+52)	GG(1)/AA+AG(26+19)	0.37(0.04–3.22)	0.370	GG(6)/AA+AG(25+27)	GG(3)/AA+AG(74+51)	0.17(0.04–0.74)	**0.018**
**BDNF-11p13**									
rs56164415	Dom	AA+AG(9+45)/GG(69)	AA+AG(3+13)/GG(30)	0.68(0.32–1.41)	0.297	AA+AG(2+9)/GG(47)	AA+AG(9+38)/GG(81)	2.63(1.31–5.29)	**0.007**
	Rec	AA(9)/GG+AG(69+45)	AA(3)/GG+AG(30+13)	0.85(0.21–3.46)	0.825	AA(2)/GG+AG(47+9)	AA(9)/GG+AG(81+38)	2.22(0.43–11.36)	0.338
**Risperidone group (n = 260)**									
**rsID**	**Test**	**Low severity group (n = 115)**				**High severity group (n = 145)**			
		**Complete responders**	**Incomplete responders**	**OR(95%CI)**	**P-value**	**Complete responders**	**Incomplete responders**	**OR(95%CI)**	**P-value**
**SLC6A3-5p15.3**									
rs2550956	Dom	TT+CT(4+33)/CC(43)	TT+CT(4+14)/CC(17)	1.31(0.56–3.07)	0.539	TT+CT(5+13)/CC(25)	TT+CT(11+49)/CC(42)	1.8(0.84–3.88)	0.132
	Rec	TT(4)/CC+CT(43+33)	TT(4)/CC+CT(17+14)	3.54(0.78–16.19)	0.103	TT(5)/CC+CT(25+13)	TT(11)/CC+CT(42+49)	0.79(0.22–2.85)	0.718
rs6347	Dom	GG+AG(2+22)/AA(56)	GG+AG(0+6)/AA(29)	0.52(0.18–1.47)	0.215	GG+AG(2+15)/AA(26)	GG+AG(0+18)/AA(84)	0.26(0.11–0.63)	**0.003**
	Rec	GG(2)/AA+AG(56+22)	GG(0)/AA+AG(29+6)	0(0–0)	0	GG(2)/AA+AG(26+15)	GG(0)/AA+AG(84+18)	0(0–0)	0
**PIP5K2A-10p12.2**									
rs10828317	Dom	CC+CT(2+23)/TT(55)	CC+CT(1+8)/TT(26)	0.96(0.37–2.5)	0.938	CC+CT(2+19)/TT(22)	CC+CT(1+27)/TT(74)	0.38(0.17–0.84)	**0.017**
	Rec	CC(2)/TT+CT(55+23)	CC(1)/TT+CT(26+8)	3.32(0.23–47.58)	0.378	CC(2)/TT+CT(22+19)	CC(1)/TT+CT(74+27)	0.15(0.01–1.77)	0.132
rs11013052	Dom	AA+AC(3+23)/CC(54)	AA+AC(1+11)/CC(23)	1.19(0.47–3)	0.706	AA+AC(4+19)/CC(20)	AA+AC(1+24)/CC(77)	0.26(0.12–0.59)	**0.001**
	Rec	AA(3)/CC+AC(54+23)	AA(1)/CC+AC(23+11)	0.8(0.08–8.34)	0.849	AA(4)/CC+AC(20+19)	AA(1)/CC+AC(77+24)	0.07(0.01–0.73)	**0.026**
rs1409396	Dom	CC+CT(11+40)/TT(29)	CC+CT(5+16)/TT(14)	0.96(0.39–2.35)	0.934	TT+CT(7+25)/CC(11)	CC+CT(8+47)/TT(47)	0.26(0.11–0.66)	**0.004**
	Rec	CC(11)/TT+CT(29+40)	CC(5)/TT+CT(14+16)	1.09(0.33–3.54)	0.889	TT(7)/CC+CT(11+25)	CC(8)/TT+CT(47+47)	0.21(0.07–0.59)	**0.003**
rs2296624	Dom	AA+AG(2+22)/GG(56)	AA+AG(1+8)/GG(26)	0.98(0.38–2.54)	0.960	AA+AG(2+18)/GG(23)	AA+AG(1+25)/GG(76)	0.38(0.17–0.86)	**0.021**
	Rec	AA(2)/GG+AG(56+22)	AA(1)/GG+AG(26+8)	3.32(0.23–47.58)	0.378	AA(2)/GG+AG(23+18)	AA(1)/GG+AG(76+25)	0.15(0.01–1.77)	0.132
rs7094131	Dom	CC+CT(2+26)/TT(52)	CC+CT(1+9)/TT(25)	0.8(0.32–1.99)	0.632	CC+CT(2+23)/TT(18)	CC+CT(1+33)/TT(68)	0.31(0.14–0.69)	**0.004**
	Rec	CC(2)/TT+CT(52+26)	CC(1)/TT+CT(25+9)	3.32(0.23–47.58)	0.378	CC(2)/TT+CT(18+23)	CC(1)/TT+CT(68+33)	0.15(0.01–1.88)	0.143
rs746203	Dom	GG+AG(5+35)/AA(40)	GG+AG(1+11)/AA(23)	0.53(0.22–1.28)	0.158	GG+AG(5+20)/AA(18)	GG+AG(1+42)/AA(59)	0.48(0.22–1.05)	0.068
	Rec	GG(5)/AA+AG(40+35)	GG(1)/AA+AG(23+11)	0.57(0.06–5.72)	0.632	GG(5)/AA+AG(18+20)	GG(1)/AA+AG(59+42)	0.07(0.01–0.65)	**0.019**
**BDNF-11p13**									
rs56164415	Dom	AA+AG(5+27)/GG(48)	AA+AG(2+9)/GG(24)	0.67(0.27–1.64)	0.377	AA+AG(0+7)/GG(36)	AA+AG(9+29)/GG(64)	3.31(1.27–8.66)	**0.015**
	Rec	AA(5)/GG+AG(48+27)	AA(2)/GG+AG(24+9)	0.79(0.14–4.56)	0.793	AA(0)/GG+AG(36+7)	AA(9)/GG+AG(64+29)	0(0–0)	0

Footnote: OR, Odds ratio; CI, Confidence interval.

a P-values and Odds ratio were adjusted with age, gender, duration of illness, age at onset.

b Remain significant after10,000 MaxT permutation for multiple testing (multiple testing was performed for 423 schizophrenia patients not for sub-groups).

Out of nine polymorphisms, eight SNPs (two of *SLC6A3* and six of *PIP4K2A*) with p<0.05 were selected for haplotype analyses to examine their role in antipsychotics response. Remaining polymorphism rs56164415 was from *BDNF* gene and hence haplotype could not be constructed. Information for identified haplotypes is provided in Table 4. Five haplotypes were observed in 423 schizophrenia patients, among them, six marker ‘ATTGCT’ haplotype (rs746203-rs10828317-rs7094131-rs2296624-rs11013052-rs1409396) of *PIP4K2A* gene showed significant association between CR (0.44) and IR (0.64) in HSG (p-value<0.002, OR = 2.05, 95%-CI = 1.26–3.33). In addition, this haplotype was associated with drug response in atypical monotherapy and risperidone subgroups and the association remained significant after applying Bonferroni corrections (Table 5). Further, this haplotype was examined in diplotype combination (ATTGCT/ATTGCT) and found to be significantly associated with incomplete antipsychotic response in patients with high severity (corrected p-value <0.0027; OR 3.37, 95%-CI = 1.51–8.28) (Table 6). Additionally, the effect of this diplotype on antipsychotics response in LSG and HSG was adjusted for age, sex, DOI and AOO in all patients, as well as in the treatment stratified groups using multivariate logistic regression analyses to negate any confounding factors. The analyses indicated independent effect of diplotype with drug response in severely ill patients among 423 samples (adjusted p-value = 0.004, OR = 3.19, 95%-CI = 1.46–6.98), 355 atypical monotherapy (adjusted p-value = 0.004, OR = 3.66, 95%-CI = 1.51–8.88) and 260 risperidone subgroup (adjusted p-value = 0.007, OR = 4.75, 95%-CI = 1.53–14.72). Receiver operating characteristic curve (ROC) clearly demonstrated that area under the curve was 6% more in HSG (0.6, 95%-CI = 0.54–0.65) than LSG (0.54; 95%-CI = 0.46–0.61) (Table 7). These results suggested an impact of this diplotype was larger in high severity patient than in patients with low severity indicating its role in therapeutic outcome.

**Table 4:** Haplotype distribution and their association analysis with drug response in schizophrenia patients (n = 423).

Groups	Gene	SNPs	Haplotype	Number (frequency)		OR (95%CI)	P-value
				CR	IR		
**Low Severity**	*SLC6A3*	rs6347|rs2550956	AT	47 (0.17)	27 (0.26)	1.91 (0.05–3.43)	0.021
			AC	196 (0.69)	59 (0.59)	0.52 (0.29–0.96)	0.021
	*PIP4K2A*	rs746203|rs10828317|rs7094131|rs2296624|rs11013052|rs1409396	GCCAAC	32 (0.12)	14 (0.14)	1.12 (0.52–2.31)	0.742
			ATTGCC	46 (0.17)	14 (0.14)	0.72 (0.34–1.43)	0.327
			ATTGCT	136 (0.5)	57 (0.56)	1.17 (0.67–2.07)	0.567
**High Severity**	*SLC6A3*	rs6347|rs2550956	AT	21 (0.17)	86 (0.27)	1.73 (0.99–3.14)	0.044
			AC	86 (0.61)	203 (0.61)	0.58 (0.32–1.01)	0.044
	*PIP4K2A*	rs746203|rs10828317|rs7094131|rs2296624|rs11013052|rs1409396	GCCAAC	25 (0.2)	46 (0.15)	0.62 (0.34–1.13)	0.091
			ATTGCC	26 (0.21)	43 (0.13)	0.55 (0.31–0.99)	0.029
			ATTGCT	53 (0.44)	190 (0.64)	2.05 (1.26–3.33)	0.002^a^

**Footnote:** SNPs with P<0.05 were included for haplotype analysis, Haplotype with minimum frequency less than 0.10 were excluded from the study,

a P-values less than threshold value of α which is equal to 0.05/18(n) = 0.0027, where n is number of test for haplotype association analysis.

CR, Complete responders; IR, Incomplete responders; OR, Odds ratio; CI, Confidence interval.

**Table 5:** Association analysis of haplotype with drug response in atypical monotherapy (n = 355) and risperidone (n = 260) group.

Groups	Genes	SNPs	Haplotype	Number (frequency)		OR (95%CI)	P-value
				CR	IR		
**Atypical monotherapy group**							
Low severity	*SLC6A3*	rs6347|rs2550956	AC	167 (0.80)	52 (0.67)	0.50 (0.27–0.94)	0.019
	*PIP4K2A*	rs746203|rs10828317|rs7094131|rs2296624|rs11013052|rs1409396	ATTGCT	49 (0.49)	115 (0.54)	0.87 (0.48–1.58)	0.631
High severity	*SLC6A3*	rs6347|rs2550956	AC	52 (0.81)	159 (0.69)	0.73 (0.37–1.40)	0.319
	*PIP5K2A*	rs746203|rs10828317|rs7094131|rs2296624|rs11013052|rs1409396	ATTGCT	44 (0.43)	151 (0.64)	2.15 (1.25–3.70)	0.003
**Risperidone group**							
Low severity	*SLC6A3*	rs6347|rs2550956	AC	107 (0.80)	44 (0.69)	0.56 (0.27–1.16)	0.086
	*PIP4K2A*	rs746203|rs10828317|rs7094131|rs2296624|rs11013052|rs1409396	ATTGCT	72 (0.49)	38 (0.54)	1.38 (0.67–2.88)	0.347
High severity	*SLC6A3*	rs6347|rs2550956	AC	54 (0.81)	127 (0.68)	0.52 (0.24–1.06)	0.055
	*PIP4K2A*	rs746203|rs10828317|rs7094131|rs2296624|rs11013052|rs1409396	ATTGCT	29 (0.39)	121 (0.63)	2.46 (1.30–4.63)	0.002^a^

**Footnote:** SNPs with P<0.05 were included for haplotype analysis, Haplotype with minimum frequency less than 0.10 were excluded from the study.

a P-values less than threshold value of α which is equal to 0.05/18(n) = 0.0027, where n is number of test for haplotype association analysis.

CR, Complete responders; IR, Incomplete responders; OR, Odds ratio; CI, Confidence interval.

**Table 6:** Distribution of diplotype, ATTGCT/ATTGCT (*PIP4K2A*) and its association analysis with drug response in schizophrenia patients (n = 423), atypical monotherapy (n = 355) and risperidone group (n = 260).

Groups	SNPs	Number (frequency)		OR (95%CI)	P-value
		CR	IR		
**Schizophrenia patients**					
Low severity	rs746203|rs10828317|rs7094131|rs2296624|rs11013052|rs1409396	33 (0.67)	16 (0.32)	1.51 (0.69–3.22)	0.252
High severity	rs746203|rs10828317|rs7094131|rs2296624|rs11013052|rs1409396	9 (0.14)	55 (0.85)	3.37 (1.51–8.28)	0.001^a^
**Atypical monotherapy group**					
Low severity	rs746203|rs10828317|rs7094131|rs2296624|rs11013052|rs1409396	28 (0.68)	13 (0.31)	1.34 (0.57–3.04)	0.458
High severity	rs746203|rs10828317|rs7094131|rs2296624|rs11013052|rs1409396	7 (0.14)	43 (0.86)	3.6 (1.45–10.15)	0.002^a^
**Risperidone group**					
Low severity	rs746203|rs10828317|rs7094131|rs2296624|rs11013052|rs1409396	16 (0.59)	11 (0.40)	1.83 (0.66–4.90)	0.18
High severity	rs746203|rs10828317|rs7094131|rs2296624|rs11013052|rs1409396	4 (0.10)	35 (0.89)	5.09 (1.62–21.01)	0.002^a^

**Footnote:** Haplotype withstand Bonferonni correction was included for diplotype analysis. Diplotype with minimum frequency less than 0.10 were ignored.

a P-values less than threshold value of α which is equal to 0.05/6(n) = 0.008, where n is number of test for diplotype association analysis.

CR, Complete responders; IR, Incomplete responders; OR, Odds ratio; CI, Confidence interval.

**Table 7:** Multivariate logistic regression analysis with incomplete antipsychotic response in schizophrenia patients stratified by treatment.

	Schizophrenia patients (n = 423)				Atypical monotherapy group (n = 355)				Risperidone group (n = 260)			
Variables	Low severity		High severity		Low severity		High severity		Low severity		High severity	
	OR (95% CI)	P -value	OR (95% CI)	P -value	OR (95% CI)	P -value	OR (95% CI)	P -value	OR (95% CI)	P -value	OR (95% CI)	P -value
**Diplotype (ATTGCT/ATTGCT)**	1.41(0.67–2.97)	0.366	3.19(1.46–6.98)	0.004^b^	1.23(0.54–2.77)	0.623	3.66(1.51–8.88)	0.004^b^	1.95(0.71–5.36)	0.193	4.75(1.53–14.72)	0.007^b^
**Sex (female)**	1.04(0.53–2.06)	0.903	1.2(0.65–2.24)	0.548	1.01(0.49–2.11)	0.971	1.18(0.59–2.33)	0.635	1.17(0.48–2.89)	0.724	1.48(0.67–3.25)	0.322
**Duration of illness (long)**	2.02(0.93–4.41)	0.078	1.93(0.99–3.74)	0.052	1.98(0.86–4.54)	0.107	1.92(0.90–4.08)	0.090	1.68(0.61–4.65)	0.317	1.79(0.73–4.39)	0.206
**Age at onset (late)**	2.58(0.83–8.02)	0.101	0.74(0.29–1.83)	0.511	2.97(0.88–10.01)	0.079	0.54(0.19–1.56)	0.255	3.93(0.98–15.77)	0.053	0.59(0.17–2.01)	0.395
**Age** a	1.08(0.83–8.03)	0.046^b^	0.97(0.91–1.04)	0.381	1.09(1.00–1.19)	0.033	0.96(0.89–1.03)	0.261	1.14(1.03–1.26)	0.011^b^	0.96(0.88–1.04)	0.345
**Nagelkerke’s R^2^**	2.98%		6.22%		3.21%		7.47%		7.07%		8.98%	
**Sensitivity**	1		0.61		1		0.67		0.98		0.73	
**Specificity**	0		0.62		0		0.61		0		0.60	
**Positive Predictive Value**	0.74		0.40		0.73		0.43		0.70		0.42	
**Negative Predictive Value**	0		0.80		0		0.81		0		0.84	
**Correctly classified**	0.74		0.62		0.73		0.63		0.69		0.64	
**ROC area (SE; 95%CI)**	0.54 (0.04; 0.46 – 0.61)		0.60 (0.03; 0.55–0.66)		0.53 (0.04; 0.45–0.60)		0.61 (0.03; 0.55–0.67)		0.56 (0.05; 0.47–0.65)		0.63 (0.03; 0.56–0.69)	

Footnote- OR, Odds ratio; ROC, Receiver operating characteristic; SE, Standard error; CI, Confidence interval.

a Taken as continuous variable.

b Significant p-value.

#### Epistasis analyses

**Figure 1:**
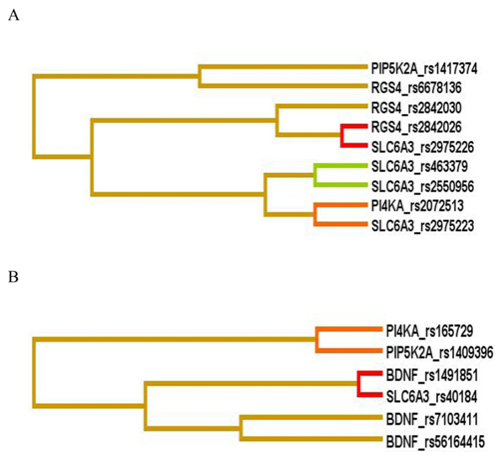
Interaction dendrogram generated from analysis of 53 polymorphisms by MDR method in patients with (A) low severity and (B) high severity of illness. A red or orange line indicates synergistic relationship; golden line represents additivity and blue or green shows redundancy. Short length arm of dendrogram indicates strong interaction. Footnote: MDR-Multifactor dimensionality reduction.

**Figure 2:**
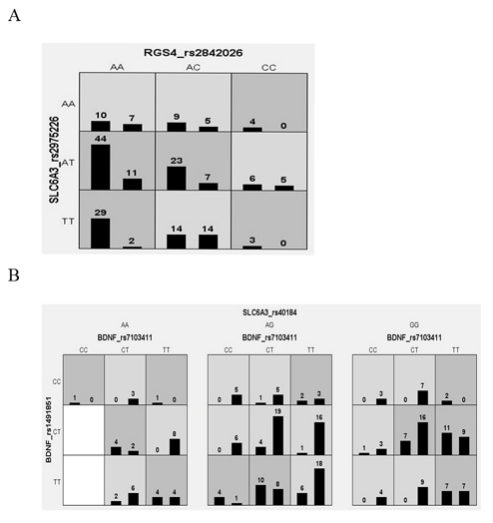
Distribution of high risk (dark shaded) and low risk (light shaded) genotypes of best models among the studied polymorphisms. Left bars illustrates distribution of complete responders and right bars shows incomplete responders for each genotype combination in (A) low severity and (B) high severity of illness patients with schizophrenia.

The MDR analysis was performed in all 423 patients to further investigate gene-gene interactions between CR and IR for both severity groups. Overall best models were *RGS4*_rs2842026-*SLC6A3*_rs2975226 and *BDNF*_rs7103411-*BDNF*_rs1491851-*SLC6A3*_rs40184 with CVC of 7/10 and p-value<0.0001 in LSG and HSG patients respectively (Table 8). It was observed that in LSG, patients with AA genotype of rs2842026 in combination with AA and AT genotypes of rs2975226 conferred 4.09 times higher risk for responsiveness towards antipsychotic treatment (OR = 4.09, 95%-CI = 2.09–8.02), while HSG patients, AG and GG genotype of rs40184 in combination with CT and TT genotype of rs7103411 and CT and TT genotypes of rs1491851 posed lower risk for non responsiveness towards antipsychotic response (OR = 7.91, 95%-CI = 4.08–15.36) (Fig. 1 and 2). These results support strong synergistic effect between these polymorphisms making an individual susceptible to incomplete antipsychotics response.

**Table 8:** Statistical comparison of different models evaluated for 53 polymorphisms with antipsychotic response by MDR analysis.

	Low severity group (n = 193)			High severity group (n = 230)		
Parameters	*RGS4*_rs2842026-*SLC6A3*_rs2975226	*PIP4K2A*_rs1417374-*RGS4*_rs2842030-*SLC6A3*_rs2550956	*PI4KA*_rs2072513-*RGS4*_rs6678136-*SLC6A3*_rs463379-*SLC6A3*_rs2975223	*BDNF*_56164415-*PIP4K2A*_rs1409396	*BDNF*_rs7103411-*BDNF*_rs1491851-*SLC6A3*_rs40184	*BDNF*_rs7103411-*BDNF*_rs1491851-*PI4KA*_rs165729-*SLC6A3*_rs40184
**CV Consistency**	**7/10**	**2/10**	**4/10**	**6/10**	**7/10**	**3/10**
**Testing Accuracy**	0.67	0.74	0.83	0.67	0.74	0.82
**Sensitivity**	0.73	0.75	0.73	0.69	0.78	0.87
**Specificity**	0.61	0.69	0.92	0.64	0.69	0.74
**OR (95%CI)**	4.09 (2.09–8.02)	6.44 (3.19–12.99)	32.16 (10.85–95.29)	4.12 (2.25–7.57)	7.91 (4.08–15.36)	18.73 (8.55–41.04)
**χ^2^ (P-value)**	18.02 (p<0.0001)	30.22 (p<0.0001)	65.11 (p<0.0001)	22.26 (p<0.0001)	42.93 (p<0.0001)	71.98 (p<0.0001)

Footnote- MDR, Multifactor dimensionality reduction; OR, Odds Ratio; CI, Confidence Interval.

## Discussion

**Figure 3:**
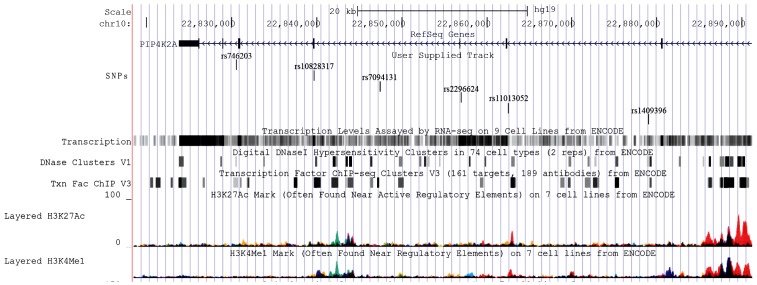
Identification of regulatory potential of six marker haplotype using ENCODE data from UCSC genome browser.

Single nucleotide variations are known to account for 90% of genetic variability, hence, we examined their effect on antipsychotic response in schizophrenia patients with variable degree of severity. Among 53 SNPs investigated from five genes, we observed TT genotype of rs1409396 (*PIP4K2A*) was significantly associated with incomplete antipsychotic response in severely ill patients in 423 schizophrenia patients as well as in subgroups. This polymorphism is present in intron 4 at conserved domain for binding of CLOCK:BMAL transcription factor and ‘T’ allele is predicted to alter its binding site. This transcription factor widely participates in responses to environmental contaminants, hypoxia, neurogenesis, synaptic plasticity and circadian regulation [42]. In addition, nominal association was detected in major alleles of five variants from *PIP4K2A* (rs10828317, rs11013052, rs2296624, rs7094131, rs746203) in severely ill patients who had incomplete response towards antipsychotic drugs. Furthermore, diplotype with major allele ‘A-T-T-G-C-T’ of six variants from *PIP4K2A* gene (rs746203-rs10828317-rs7094131-rs2296624-rs11013052-rs1409396) was found to be significantly associated which suggested that it may influence treatment non-responsiveness however, its actual effects should be elucidated by in-vitro and in-vivo approaches. As predicted by ENCODE data (RegulomeDb and HaploReg) variants constituting this diplotype have putative transcription factor binding sites which emphasizes that diplotype may have important biological consequences (Figure 3). Polymorphism rs11013052 was predicted to alter the motifs of ERalpha-a_disc4, Pbx3_disc2 which may influence the expression of estrogen alpha receptor and pre-B-cell leukemia homeobox 3. The rs7094131 was found to be present in conserved region of intron 6 and may alter binding sites for various transcription factors whereas rs746203 was present in expression quantitative trait loci and DNAseI hypersensitive site. One non-synonymous variation rs1082817 was known to down-regulate the synthesis of PIP_2_ in *Xenopus* system and completely abolish the kinase activity [43]. However, recently, investigators did not find significant difference in mutated-enzyme activity with wild-type enzyme [16]. Over-representation of this diplotype in severely ill incomplete responders is consistent with consequence of altered phosphoinositide pathway in schizophrenia symptomatology and drug response through modulation of genes comes to play after drug-target interaction.

The PIP4K2A catalyses phosphorylation of PI(5)P (phosphatidylinosito-phosphate) and lead to formation of PI(4,5)P_2_ (PIP_2_) which further hydrolyzed by the action of phospholipase C into second messenger inositoltrisphosphate and diacylglycerol [44]–[46]. PIP_2_ was shown to be involved in the regulation of secretion, cell proliferation, differentiation, motility and, synaptic vesicle cycle [47]. It is a key player of membrane transduction of neurotransmitter signals, intracellular signaling, which may be impaired in schizophrenia. This pathway was proven to be potential target for lithium action for treatment of bipolar disorder [48]. *PIP4K2A* controls the functioning of KCNQ channels that further suppress the basal activity of dopaminergic neurons and their firing in substantia nigra and ventral tegmental area of brain [43]. Moreover, *PIP4K2A* stimulate excitatory amino acid transporter, EAAT3 which clears glutamate from synaptic cleft. Impaired activity of PIP4K2A decreases EAAT3 activity and is expected to foster glutamate toxicity in schizophrenia patients [49].

To the best of our knowledge, this is the first study that examined *PIP4K2A* for antipsychotic response. Interestingly, we found similar frequencies of *PIP4K2A* variants upon comparison among all schizophrenia cases, patients in LSG and HSG with controls. We failed to detect association of *PIP4K2A* in case-control analyses, irrespective of disease severity (data not shown). Our study further corroborated to existing evidence for involvement of *PIP4K2A* gene in schizophrenia disease and it was in conformity with other association studies where, major alleles of variants were found to be over-represented in disease individuals as compared to control [17], [50], [51]. Phased haplotype and diplotype are more informative and have high statistical power for an association than single SNP association because SNP do not arise independently; they have intrinsic dependency with one another due to their introduction in genealogical process [52]. Twelve locus haplotype of *PIP4K2A* ‘G-A-T-A-A-A-G-DEL-C-G-A-G’ (rs7081744-rs943190-rs10828317-rs10828316-rs10430590-rs746203-rs1053454-rs3215826-rs8341-rs2364115-rs214836-rs1536332) showed over-transmission of major alleles to schizophrenia affected offspring of German and Israeli populations [17]. Similarly, major allele ‘T’ of rs10828317 found to be associated with deficit schizophrenia and non-deficit schizophrenia patients in Dutch population [50]. Major allele ‘C’ of another intronic variant rs11013052 was found to be over-transmitted in offspring affected with schizophrenia in Indonesian population [51]. On the contrary, a three-locus haplotype C-G-A (rs10828317-rs746203-rs8341) of minor alleles was found to be associated in Chinese population [53]. Further, under-representation of common alleles preferentially in schizophrenia affected females was detected in Irish population [15]. The CC genotype (C is minor allele) of rs10828317 was found to be associated in Russian population, however another study failed to detect association for *PIP4K2A* variant in German population [54], [55]. These studies provide additional evidence that defective phosphoinositide signaling contributes to schizophrenia etiology. We analyzed frequency of six variants of *PIP4K2A* gene across the globe and found similar frequency distribution as in study population (Table S4 in File S1). However, in African and Japanese population major allele of two variants (rs7094131; rs746203) were found to be under-represented. Intra-locus heterogeneity (different variants within the same gene) could be one of the reason for this variable allele frequency and *PIP4K2A* best explains this phenomenon. This heterogeneity has been proposed as a major factor for the inconsistencies among the large population association test. Interestingly, these SNPs were not in LD with other remaining associated SNPs in 1000 genome populations as well as in our population. This indicated, that combination of these variants, if present may influence antipsychotic response in severely ill patients. Thus, we expect that, these polymorphisms may have role in gene expression and regulation and hence important for pharmacogenomic and other epidemiology studies. The largest prospective observational SOHO study suggested that it is important to assess treatment impact separately in differentially affected patients according to severity and type of schizophrenia symptoms [56]. Further, Chen and colleagues examined the magnitude of antipsychotic response to predict non-response as ‘not minimally improved’, ‘not much improved’ and ‘not remitted’ in schizophrenia patients classified as moderately to severely ill and less than moderately ill [3]. Our data further add to existing evidences indicating disease severity can modulate antipsychotic response. Thus, it would be helpful if analyses in future association studies consider clinical factors including disease severity separately for identification of predictive genetic markers that accurately defines treatment response.

In addition to *PIP4K2A*, we also observed nominal significant association for rs56164415 (C270T, *BDNF*), rs2550956 and rs6347 (*SLC6A3*) with antipsychotics response. Although similar distribution within other populations was observed for polymorphisms from *RGS4* and *PI4KA* gene p-value <0.05 did not allow a genotype-phenotype correlation. This indicates that association difference may be attributable to genetic heterogeneity among different ethnic groups. Inconsistent associations were reported for polymorphism rs56164415 with antipsychotic response as it was not associated in Finnish population whereas haplotype 230bp-C-A (GT_n_ repeat-rs56164415-rs6265) was associated with risperidone response in Han-Chinese schizophrenia patients [57], [58]. An updated meta-analysis revealed ‘T’ allele of rs56164415 associated with schizophrenia in East Asian population but not in Caucasian [59]. Also, rs56164415 predicted to be present in CpG island (high GC content-63.6%) can influence gene expression and post-transcriptional regulation of gene expression [60]. Other than this, rs6265 and rs11030104 were extensively studied in context of disease and antipsychotic response but, so far associations are inconclusive [61], [62]. Reduced *BDNF* levels were observed in medicated and drug naive schizophrenia patients as indicated by meta-analysis with unexplained population heterogeneity [63]. In a recent study, authors detected overall facilitation of Bdnf expression in aripiprazole-treated animals and this provide novel information regarding the mechanisms that aripiprazole may regulate brain function through *BDNF* and could contribute to improvement of schizophrenia associated neuroplastic [64]. *SLC6A3* variants rs2550956 and rs6347 were associated with schizophrenia [65]–[68]. These multiple genetic variants might have strong synergistic effects beyond their weak individual effect. This indicates that inappropriate dopamine efflux may be an unexplored mechanism that modulates the therapeutic actions of existing dopamine and serotonin receptor antagonist drugs. Even though some candidates from pharmacogenetic studies were replicated and seemed to be promising, still to date, there are no predictive marker developed that are commonly accepted to assess antipsychotic response. Heterogeneity among genetic architecture, populations and phenotype, different diagnostic criteria, gene-gene and gene-environmental interactions could be reasons for the failure of pharmacogenomic studies. In present study, MDR results suggested interaction between *RGS4*_rs2842026-*SLC6A3*_rs2975226 in schizophrenia patients of LSG and between *BDNF*_rs7103411-*BDNF*_rs1491851-*SLC6A3*_rs40184 in severely ill patients for their inadequate treatment response. These epigenetic effects could best explain descriptive phenotypes such as complete responders and incomplete responders, variable degree of severity. This also reflects pleiotropic effects of variants which may have important implications for drug discovery.

Observed associations in the present study are true for several reasons as we applied stringent quality control procedures for inclusion of SNP, performed diplotype analysis with appropriate multiple test corrections and study participants were genotyped with microsatellite markers for testing homogeneity. However, results of the present study need to be interpreted cautiously because of three main limitations. First, limited number of candidate genes was analyzed. Secondly, our sample pool was in-sufficient for testing association of rare and uncommon variants. However, common variants have comparatively high power and accounts for variability in endophenotype for a disease [69]. The third limitation was lack of replication of results in another cohort. Studies with larger coverage and gene mapping with comprehensive analysis and replication are required to support and provide biological insight into disease pathogenesis and drug response mechanism.

In conclusion, study highlights the importance of *PIP4K2A* as crucial signaling partner in drug induced signaling and its relationship with clinical characteristics of disease as we identified a risk diplotype of *PIP4K2A* which may predict antipsychotic incomplete response in severely ill schizophrenia patients. The identified marker may be used for early detection of patients who are not responding to their current treatment and may require change for optimum therapeutic regimen and can be exploited for novel therapeutics as add-on to antipsychotics for betterment of the patients. However, study merits replication of results in a larger population followed by functional validation. The complex etiology of schizophrenia merits the consideration of both genetic and epigenetic systems and the meticulous experiment designs that unravel the underlying mechanisms conferring accountability for schizophrenia and development of new efficacious therapies.

## Supporting Information

Figure S1 Summary plot of estimates of *Q*. Single vertical line represent each individual. Line broken into *K* colored segments with lengths proportional to each of the *K* inferred clusters including data of 441 neutral markers.
(JPG)

File S1 **Table S1a,** Genetic homogeneity test for stratification by comparing distribution of 10 unlinked microsatellites markers between schizophrenia patients (n = 482) and healthy controls (n = 230). Footnote: χ^2^-Chi Sqaure; df-Degree of freedom. **Table S1b,** Genetic homogeneity test for stratification by comparing distribution of 441 bi-allelic neutral markers between schizophrenia patients (n = 482) and healthy controls (n = 215). **Table S2,** Number statistics of prioritized SNPs for five genes. Footnote: SNPs- Single nucleotide polymorphism; HWE- Hardy-Weinberg equilibrium; LD- Linkage disequilibrium; MAF- Minor allele frequency; r^2^- correlation coefficient, measurement of LD. **Table S3,** Details and allele frequencies of polymorphisms in schizophrenia patients (n = 482) and controls (n = 230). Footnote: ^a^From RegulomeDB and HaploReg; ^b^Major allele/minor allele; ^c^Minor allele frequency. **Table S4,** Global frequency and world-wide frequency of major allele six drug response associated polymorphisms of PIP4K2A typed in 1000 genomes. Footnote: N-number of individuals; Major allele and major allele frequencies were provided in this table.
(DOC)
