# Supplementary figures and images for: Genetic Variations of PIP4K2A Confer Vulnerability to Poor Antipsychotic Response in Severely Ill Schizophrenia Patients
Source: PLoS One. 2014 Jul 15;9(7):e102556. doi: 10.1371/journal.pone.0102556 (PMC4099378; doi:10.1371/journal.pone.0102556)

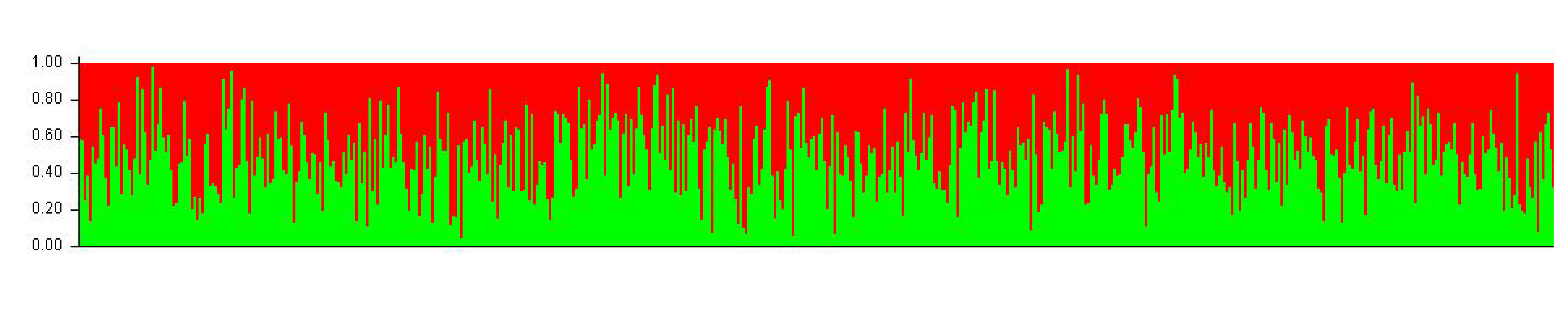

Supplement: Figure S1 — Summary plot of estimates of Q. Single vertical line represent each individual. Line broken into K colored segments with lengths proportional to each of the K inferred clusters including data of 441 neutral markers. (JPG) [file pone.0102556.s001.jpg]
