# Supplementary material for: Genetic Variations of PIP4K2A Confer Vulnerability to Poor Antipsychotic Response in Severely Ill Schizophrenia Patients
Source: PLoS One. 2014 Jul 15;9(7):e102556. doi: 10.1371/journal.pone.0102556 (PMC4099378; doi:10.1371/journal.pone.0102556)
Supplement: File S1 — Table S1a, Genetic homogeneity test for stratification by comparing distribution of 10 unlinked microsatellites markers between schizophrenia patients (n = 482) and healthy controls (n = 230). Footnote: χ2-Chi Sqaure; df-Degree of freedom. Table S1b, Genetic homogeneity test for stratification by comparing distribution of 441 bi-allelic neutral markers between schizophrenia patients (n = 482) and healthy controls (n = 215). Table S2, Number statistics of prioritized SNPs for five genes. Footnote: SNPs- Single nucleotide polymorphism; HWE- Hardy-Weinberg equilibrium; LD- Linkage disequilibrium; MAF- Minor allele frequency; r2- correlation coefficient, measurement of LD. Table S3, Details and allele frequencies of polymorphisms in schizophrenia patients (n = 482) and controls (n = 230). Footnote: aFrom RegulomeDB and HaploReg; bMajor allele/minor allele; cMinor allele frequency. Table S4, Global frequency and world-wide frequency of major allele six drug response associated polymorphisms of PIP4K2A typed in 1000 genomes. Footnote: N-number of individuals; Major allele and major allele frequencies were provided in this table. (DOC) [file pone.0102556.s002.doc]

**Table S1a: Genetic homogeneity test for stratification by comparing distribution of 10 unlinked microsatellites markers between schizophrenia patients (n=482) and healthy controls (n=230)**

| **S. No.** | **Marker ID** | **Comparison between patients and controls** | | |
| --- | --- | --- | --- | --- |
|  |  | **χ^2^** | **df** | **p-value** |
| 1 | D2S2216 | 4.008 | 7 | 0.76 |
| 2 | D2S2259 | 12.53 | 8 | 0.091 |
| 3 | D2S162 | 9.565 | 15 | 0.961 |
| 4 | D2S2330 | 12.49 | 11 | 0.407 |
| 5 | D4S391 | 11.84 | 9 | 0.051 |
| 6 | D4S412 | 9.456 | 9 | 0.662 |
| 7 | D7S519 | 19.90 | 11 | 0.299 |
| 8 | D7S2465 | 12.02 | 9 | 0.306 |
| 9 | D7S517 | 8.708 | 7 | 0.282 |
| 10 | D7S530 | 9.26 | 9 | 0.647 |
| **Sum χ^2^ (df); p-value** | | **109.79 (95); 0.142** | | |

Footnote: χ^2^, Chi Sqaure; df, Degree of freedom

**Table S1b: Genetic homogeneity test for stratification by comparing distribution of 441 bi-allelic neutral markers between schizophrenia patients (n=482) and healthy controls (n=215)**

| **SNP_ID** | **Chi-square** | **P-value** |
| --- | --- | --- |
| rs619729 | 1.943 | 0.096 |
| rs3845520 | 0.004 | 0.645 |
| rs6679217 | 1.229 | 0.172 |
| rs409914 | 1.135 | 0.399 |
| rs10489163 | 0.212 | 0.442 |
| rs1007266 | 0.119 | 0.9 |
| rs787494 | 0.739 | 0.69 |
| rs1022013 | 0.035 | 0.836 |
| rs1389789 | 0.555 | 0.744 |
| rs10493708 | 0.052 | 0.906 |
| rs2390734 | 1.501 | 0.307 |
| rs7547180 | 0.456 | 0.596 |
| rs1335745 | 0.016 | 0.629 |
| rs1930211 | 0.007 | 0.813 |
| rs970860 | 0 | 0.744 |
| rs1775700 | 0.762 | 0.4 |
| rs6428891 | 2.476 | 0.121 |
| rs10489824 | 1.003 | 0.537 |
| rs2789417 | 2.586 | 0.132 |
| rs7530581 | 1.484 | 0.408 |
| rs10489363 | 0.27 | 0.724 |
| rs3766642 | 0.778 | 0.679 |
| rs4652621 | 1.639 | 0.211 |
| rs400172 | 0.801 | 0.663 |
| rs1578243 | 0.969 | 0.387 |
| rs10494679 | 0.615 | 0.465 |
| rs10494696 | 0.707 | 0.697 |
| rs3001161 | 0.003 | 0.859 |
| rs487681 | 0.004 | 0.927 |
| rs2185781 | 0.002 | 0.618 |
| rs6670438 | 0.081 | 0.906 |
| rs2012130 | 0.021 | 0.643 |
| rs10494870 | 0.035 | 0.279 |
| rs10494898 | 2.15 | 0.26 |
| rs10494934 | 0.031 | 0.069 |
| rs1415249 | 1.256 | 0.059 |
| rs921322 | 0.107 | 0.488 |
| rs1569120 | 0.017 | 0.9 |
| rs340722 | 2.826 | 0.264 |
| rs964864 | 0.726 | 0.679 |
| rs10495716 | 0.278 | 0.436 |
| rs780102 | 1.407 | 0.433 |
| rs10495766 | 0.001 | 0.594 |
| rs1405085 | 0.037 | 0.093 |
| rs9309149 | 0.232 | 0.878 |
| rs720201 | 0.31 | 0.738 |
| rs9309464 | 0.301 | 0.725 |
| rs205638 | Non polymorphic | |
| rs10496207 | 0.738 | 0.724 |
| rs1993694 | 0.95 | 0.41 |
| rs4832311 | 0.013 | 0.567 |
| rs10496325 | 1.028 | 0.347 |
| rs2045057 | Non polymorphic | |
| rs10496353 | 0.008 | 0.886 |
| rs10496412 | 0.079 | 0.831 |
| rs2561445 | 1.583 | 0.115 |
| rs1879494 | 3.75 | 0.143 |
| rs10496431 | 1.59 | 0.497 |
| rs724496 | 0.006 | 0.901 |
| rs9308769 | 1.179 | 0.561 |
| rs10496562 | 1.214 | 0.553 |
| rs10496671 | 0.133 | 0.358 |
| rs7574678 | 2.365 | 0.323 |
| rs1371031 | 1.107 | 0.085 |
| rs155594 | 1.287 | 0.501 |
| rs411291 | 0.011 | 0.558 |
| rs2461763 | 0.304 | 0.791 |
| rs2885116 | 0.054 | 0.951 |
| rs6705679 | 0 | 0.711 |
| rs2368351 | 0.047 | 0.755 |
| rs826142 | 0.002 | 0.963 |
| rs4667014 | 0.97 | 0.629 |
| rs10497667 | 2.104 | 0.217 |
| rs10497681 | 1.648 | 0.144 |
| rs2356653 | 3.01 | 0.071 |
| rs10497731 | 2.105 | 0.261 |
| rs2884207 | 0.226 | 0.653 |
| rs801321 | 1.222 | 0.283 |
| rs723021 | 0.051 | 0.966 |
| rs357157 | 0.495 | 0.18 |
| rs890640 | 0.162 | 0.309 |
| rs1500415 | 0 | 0.303 |
| rs2370990 | 0.885 | 0.393 |
| rs623244 | 0.902 | 0.646 |
| rs7625092 | 3.217 | 0.005 |
| rs10510698 | Non polymorphic | |
| rs10490786 | 0.099 | 0.921 |
| rs2049777 | 0.376 | 0.669 |
| rs1533677 | 0.185 | 0.302 |
| rs10510751 | 0.18 | 0.912 |
| rs3849526 | 0.003 | 0.818 |
| rs9311694 | 1.718 | 0.435 |
| rs10511022 | 1.168 | 0.385 |
| rs1393584 | 0.098 | 0.931 |
| rs417281 | Non polymorphic | |
| rs10511091 | 2.312 | 0.043 |
| rs1377789 | 0.071 | 0.937 |
| rs9310004 | 1.988 | 0.142 |
| rs1546223 | 0.236 | 0.682 |
| rs1024055 | 2.806 | 0.223 |
| rs10511249 | 1.729 | 0.252 |
| rs1534504 | 0.008 | 0.951 |
| rs697954 | 0.005 | 0.585 |
| rs2715694 | 0.601 | 0.695 |
| rs1163349 | 0.921 | 0.56 |
| rs1406493 | 1.078 | 0.46 |
| rs10511289 | 0.119 | 0.73 |
| rs9288969 | 0.014 | 0.984 |
| rs524438 | 0.042 | 0.988 |
| rs1905441 | 0.376 | 0.533 |
| rs2896565 | 0.838 | 0.349 |
| rs10512862 | 2.307 | 0.326 |
| rs211588 | 0.04 | 0.8 |
| rs1992093 | 0.09 | 0.318 |
| rs197827 | 0.522 | 0.69 |
| rs1897127 | 0.067 | 0.677 |
| rs9283603 | 0.664 | 0.66 |
| rs1520818 | 1.063 | 0.119 |
| rs10513451 | 1.524 | 0.334 |
| rs1402731 | 0.169 | 0.523 |
| rs10513631 | 0.529 | 0.491 |
| rs3913367 | 0.92 | 0.073 |
| rs727272 | 0.102 | 0.95 |
| rs2165609 | 0.205 | 0.248 |
| rs1364593 | 0.008 | 0.658 |
| rs281817 | 0.991 | 0.485 |
| rs1355533 | 0.013 | 0.993 |
| rs726111 | 0.489 | 0.787 |
| rs10489082 | 1.013 | 0.556 |
| rs10516406 | 0.264 | 0.828 |
| rs800421 | 2.83 | 0.239 |
| rs7698037 | 0.41 | 0.697 |
| rs2110176 | 0.215 | 0.796 |
| rs1542458 | 0.497 | 0.289 |
| rs9283670 | 0.048 | 0.821 |
| rs10517072 | 0.432 | 0.674 |
| rs956275 | 0.011 | 0.828 |
| rs725264 | 0.905 | 0.288 |
| rs952938 | 2.458 | 0.22 |
| rs3923243 | 0 | 0.565 |
| rs958848 | 2.102 | 0.367 |
| rs10516808 | 0.181 | 0.484 |
| rs2172891 | 0.028 | 0.813 |
| rs280105 | 1.474 | 0.308 |
| rs6837318 | 0 | 0.551 |
| rs1960734 | 0.002 | 0.976 |
| rs7676731 | 0.014 | 0.986 |
| rs10516587 | 2.104 | 0.323 |
| rs4131450 | 0.598 | 0.721 |
| rs4131849 | 2.235 | 0.145 |
| rs306388 | 1.471 | 0.419 |
| rs1486991 | 0.049 | 0.709 |
| rs4132743 | 0.318 | 0.416 |
| rs10519370 | 0.095 | 0.364 |
| rs1995960 | 1.125 | 0.465 |
| rs12644496 | 1.52 | 0.411 |
| rs2014131 | 2.509 | 0.241 |
| rs2879979 | 0.014 | 0.782 |
| rs1903291 | 0.139 | 0.939 |
| rs10517809 | 2.051 | 0.119 |
| rs404016 | 2.196 | 0.319 |
| rs4866602 | 1.778 | 0.397 |
| rs10512744 | 1.824 | 0.14 |
| rs563624 | 1.385 | 0.437 |
| rs182855 | 0.889 | 0.603 |
| rs904747 | 0.645 | 0.158 |
| rs1553949 | 0.184 | 0.123 |
| rs2448464 | 1.922 | 0.116 |
| rs4129707 | 0.183 | 0.182 |
| rs950812 | 0.015 | 0.994 |
| rs10512876 | 0.087 | 0.185 |
| rs27246 | 0.226 | 0.617 |
| rs9292147 | 0.081 | 0.694 |
| rs1449274 | 0.221 | 0.85 |
| rs28899 | 1.216 | 0.566 |
| rs10491362 | 1.52 | 0.132 |
| rs6863354 | 0.6 | 0.402 |
| rs10514323 | 1.613 | 0.224 |
| rs1505851 | 0.016 | 0.717 |
| rs10514354 | 0.058 | 0.8 |
| rs1037796 | 0.265 | 0.763 |
| rs2311735 | 1.156 | 0.113 |
| rs1422004 | 0.252 | 0.372 |
| rs9327129 | 0.185 | 0.872 |
| rs10519720 | 0.021 | 0.44 |
| rs723187 | 2.696 | 0.272 |
| rs1510954 | 0.447 | 0.467 |
| rs248514 | 0.001 | 0.69 |
| rs17115277 | Non polymorphic | |
| rs4960299 | 2.094 | 0.305 |
| rs4130861 | 1.469 | 0.381 |
| rs823695 | 1.048 | 0.585 |
| rs998645 | 0.032 | 0.73 |
| rs10484246 | 0.412 | 0.681 |
| rs214554 | 0.822 | 0.655 |
| rs9295512 | 0.026 | 0.554 |
| rs926402 | 0.402 | 0.807 |
| rs10484551 | 1.797 | 0.267 |
| rs9296092 | 1.515 | 0.382 |
| rs6936472 | 0.067 | 0.903 |
| rs9294122 | 0.024 | 0.976 |
| rs10498847 | 0.752 | 0.283 |
| rs9294768 | 0.046 | 0.968 |
| rs4706479 | 1.312 | 0.259 |
| rs9293929 | 0.119 | 0.38 |
| rs9320598 | 0.422 | 0.805 |
| rs6912685 | 2.446 | 0.29 |
| rs10484272 | 1.847 | 0.414 |
| rs9320117 | 0.798 | 0.557 |
| rs10484274 | 0.047 | 0.934 |
| rs189410 | 0.888 | 0.579 |
| rs1114622 | 3.495 | 0.058 |
| rs10484771 | 0.206 | 0.855 |
| rs2235561 | 0.933 | 0.49 |
| rs1496113 | 2.5 | 0.19 |
| rs10485354 | 0.63 | 0.712 |
| rs7773053 | 3.482 | 0.157 |
| rs2235824 | 0 | 0.252 |
| rs909474 | 1.183 | 0.352 |
| rs836535 | 0.709 | 0.119 |
| rs6945372 | 0.99 | 0.605 |
| rs3095000 | 1.573 | 0.378 |
| rs10245794 | 0.785 | 0.29 |
| rs10228137 | 0.6 | 0.584 |
| rs10499698 | 2.619 | 0.24 |
| rs480777 | 3.394 | 0.161 |
| rs1595989 | 2.784 | 0.233 |
| rs687547 | 1.055 | 0.324 |
| rs2037490 | 3.654 | 0.108 |
| rs10499806 | 0.515 | 0.348 |
| rs2107351 | 1.422 | 0.407 |
| rs10228847 | 0.05 | 0.4 |
| rs10500055 | 0.001 | 0.721 |
| rs1594423 | 0.008 | 0.883 |
| rs3847102 | 0.035 | 0.63 |
| rs1420834 | 0.542 | 0.799 |
| rs951701 | 0.053 | 0.729 |
| rs1425723 | 1.291 | 0.553 |
| rs10503829 | 1.625 | 0.468 |
| rs2911691 | 0.21 | 0.752 |
| rs763878 | 1.176 | 0.385 |
| rs1603681 | 3.171 | 0.185 |
| rs10504162 | 0.006 | 0.264 |
| rs4333639 | 0.473 | 0.404 |
| rs1371322 | 0.037 | 0.502 |
| rs3954895 | 0.021 | 0.867 |
| rs10504487 | 0.155 | 0.734 |
| rs9298242 | 1.066 | 0.306 |
| rs1915546 | 0.197 | 0.777 |
| rs10504719 | 2.015 | 0.191 |
| rs890601 | 1.484 | 0.496 |
| rs7829511 | 0.239 | 0.822 |
| rs2930065 | 0.572 | 0.044 |
| rs996694 | 0.617 | 0.337 |
| rs1037701 | 0.08 | 0.785 |
| rs899877 | 0.672 | 0.533 |
| rs10505185 | 0.008 | 0.34 |
| rs10505461 | 0.12 | 0.513 |
| rs2318832 | 0.866 | 0.544 |
| rs1547258 | 3.042 | 0.145 |
| rs2997555 | 1.039 | 0.564 |
| rs1580540 | 1.098 | 0.591 |
| rs1408799 | 0.497 | 0.685 |
| rs966313 | 0.537 | 0.441 |
| rs3915429 | 1.621 | 0.465 |
| rs2885170 | 0.154 | 0.803 |
| rs10511874 | 1.275 | 0.325 |
| rs2376455 | 0 | 0.968 |
| rs1856190 | 0.029 | 0.132 |
| rs10511918 | Non polymorphic | |
| rs1580108 | 1.71 | 0.431 |
| rs3907292 | 0.155 | 0.894 |
| rs1933583 | 0.522 | 0.602 |
| rs10512148 | 0.091 | 0.262 |
| rs10512181 | 0.269 | 0.84 |
| rs1980888 | 0.019 | 0.814 |
| rs1608973 | 0.038 | 0.29 |
| rs2417562 | 0.489 | 0.597 |
| rs1570303 | 0.066 | 0.836 |
| rs10508225 | 1.415 | 0.288 |
| rs1036747 | 0.966 | 0.574 |
| rs10508295 | 0.523 | 0.808 |
| rs2255014 | 0.603 | 0.451 |
| rs1387837 | 0.586 | 0.497 |
| rs953093 | 0.017 | 0.94 |
| rs4529807 | 0.649 | 0.466 |
| rs869312 | 0.295 | 0.581 |
| rs10508867 | 0.332 | 0.585 |
| rs2393949 | 0 | 0.071 |
| rs10508993 | 1.064 | 0.345 |
| rs723212 | 1.008 | 0.567 |
| rs7922793 | 2.375 | 0.229 |
| rs10509767 | 0.816 | 0.181 |
| rs2901587 | 1.03 | 0.344 |
| rs1339822 | 0.21 | 0.898 |
| rs1112707 | 1.766 | 0.407 |
| rs10510041 | 0.157 | 0.443 |
| rs10510057 | 0.021 | 0.569 |
| rs2388847 | 1.113 | 0.517 |
| rs552390 | 0.16 | 0.802 |
| rs1160219 | 0.169 | 0.825 |
| rs1579020 | 0.584 | 0.637 |
| rs481554 | 0.095 | 0.729 |
| rs3898926 | 0.101 | 0.82 |
| rs7122369 | 0.447 | 0.521 |
| rs2666895 | 0.016 | 0.863 |
| rs693717 | 0.439 | 0.58 |
| rs7932437 | 0.005 | 0.64 |
| rs10501398 | 0.283 | 0.658 |
| rs1944130 | 1.258 | 0.48 |
| rs826056 | 0.263 | 0.679 |
| rs1944033 | 0.491 | 0.549 |
| rs1790542 | 0.048 | 0.366 |
| rs1986413 | 0.002 | 0.522 |
| rs1439527 | 1.354 | 0.303 |
| rs2097160 | 0.728 | 0.673 |
| rs567102 | 0.01 | 0.836 |
| rs1941382 | 0.004 | 0.907 |
| rs715797 | 1.953 | 0.305 |
| rs1921332 | 0.603 | 0.739 |
| rs2111368 | 0.487 | 0.422 |
| rs1278607 | 0.52 | 0.45 |
| rs1380120 | 0.016 | 0.984 |
| rs9315569 | 0.161 | 0.476 |
| rs7987497 | 1.775 | 0.167 |
| rs10507342 | 2.714 | 0.144 |
| rs9319336 | 0.097 | 0.908 |
| rs4943143 | 0.252 | 0.87 |
| rs9315425 | 0.528 | 0.526 |
| rs1923682 | 0.501 | 0.743 |
| rs1989252 | 1.473 | 0.037 |
| rs719614 | 0.045 | 0.402 |
| rs9316478 | 2.285 | 0.31 |
| rs768015 | Non polymorphic | |
| rs10507688 | 0.75 | 0.36 |
| rs2876745 | 0.318 | 0.797 |
| rs4287457 | 0.28 | 0.634 |
| rs9319102 | 1.108 | 0.426 |
| rs1894756 | 1.562 | 0.116 |
| rs1114967 | 0.101 | 0.356 |
| rs712486 | 0.547 | 0.74 |
| rs10483492 | 0.426 | 0.477 |
| rs1951349 | 0.029 | 0.927 |
| rs2415899 | 0.914 | 0.44 |
| rs8009114 | 0.007 | 0.022 |
| rs8019707 | 0.853 | 0.231 |
| rs8012374 | 0.033 | 0.04 |
| rs36409 | 0.008 | 0.93 |
| rs9323610 | 0.02 | 0.607 |
| rs850748 | 0.797 | 0.675 |
| rs10483888 | 0.212 | 0.414 |
| rs4904705 | 2.218 | 0.174 |
| rs1741240 | 0.04 | 0.804 |
| rs1351805 | 1.026 | 0.558 |
| rs1257282 | 2.18 | 0.235 |
| rs4128139 | 0.336 | 0.834 |
| rs1470148 | 0.346 | 0.701 |
| rs4072386 | 1.299 | 0.506 |
| rs2412822 | 1.31 | 0.225 |
| rs518222 | 3.224 | 0.134 |
| rs8032921 | 2.447 | 0.082 |
| rs1380839 | 0.429 | 0.802 |
| rs477966 | 0.325 | 0.621 |
| rs4776680 | 3.57 | 0.098 |
| rs2415141 | 1.139 | 0.08 |
| rs1079396 | 1.039 | 0.043 |
| rs10519236 | 0.336 | 0.466 |
| rs718276 | 2.065 | 0.029 |
| rs352744 | 0.65 | 0.696 |
| rs10500326 | 0.9 | 0.132 |
| rs4077730 | 1.002 | 0.195 |
| rs1544352 | 0.429 | 0.731 |
| rs2169795 | 2.307 | 0.291 |
| rs4131451 | 0.018 | 0.971 |
| rs392728 | 0.323 | 0.006 |
| rs2881599 | 1.209 | 0.503 |
| rs166152 | 3.352 | 0.188 |
| rs370159 | 0.08 | 0.861 |
| rs10518838 | 0.011 | 0.962 |
| rs10521212 | 2.249 | 0.314 |
| rs1420984 | 0.819 | 0.646 |
| rs10492810 | 2.04 | 0.36 |
| rs4238964 | 0.009 | 0.892 |
| rs1560278 | 0.395 | 0.788 |
| rs200287 | 0.003 | 0.913 |
| rs387953 | 0.002 | 0.563 |
| rs889608 | 0.573 | 0.732 |
| rs2353033 | 1.171 | 0.401 |
| rs10521125 | 0.015 | 0.164 |
| rs10491093 | 0.036 | 0.985 |
| rs9303104 | 0.001 | 0.686 |
| rs4072454 | 0 | 0.763 |
| rs899342 | 0.882 | 0.582 |
| rs2521984 | 0.189 | 0.742 |
| rs10512490 | 0.467 | 0.714 |
| rs10514981 | 0.016 | 0.618 |
| rs1512868 | 0.912 | 0.573 |
| rs2447455 | 0.016 | 0.518 |
| rs10491093 | 0.036 | 0.985 |
| rs918077 | 0.073 | 0.084 |
| rs1860351 | 1.292 | 0.463 |
| rs1426241 | 0.313 | 0.22 |
| rs333106 | 0.4 | 0.811 |
| rs10513907 | 0.118 | 0.185 |
| rs1498199 | 0.045 | 0.618 |
| rs1986750 | 3.433 | 0.082 |
| rs10502432 | 0.188 | 0.585 |
| rs7407701 | 0.41 | 0.808 |
| rs492443 | 0.006 | 0.801 |
| rs1943228 | 0.55 | 0.26 |
| rs7228560 | 3.105 | 0.204 |
| rs10515996 | 0.373 | 0.744 |
| rs4419154 | 2.564 | 0.202 |
| rs1113144 | 0.02 | 0.571 |
| rs1553422 | 1.452 | 0.301 |
| rs350825 | 0.761 | 0.472 |
| rs8109169 | 0.285 | 0.854 |
| rs2009234 | 0.688 | 0.685 |
| rs10500226 | 1.01 | 0.302 |
| rs10500284 | 1.395 | 0.502 |
| rs2037735 | 1.213 | 0.355 |
| rs3786777 | 0.004 | 0.983 |
| rs103294 | 3.896 | 0.057 |
| rs488196 | 0.741 | 0.57 |
| rs2253977 | 0.008 | 0.512 |
| rs2876032 | 0.371 | 0.303 |
| rs6086141 | 1.02 | 0.505 |
| rs10485767 | 1.485 | 0.472 |
| rs731667 | 2.382 | 0.286 |
| rs402120 | 0.036 | 0.724 |
| rs242528 | 0.013 | 0.631 |
| rs6060578 | 1.867 | 0.255 |
| rs6021120 | 0.443 | 0.734 |
| rs234623 | 1.391 | 0.128 |
| rs400590 | 0.052 | 0.695 |
| rs157740 | 0 | 0.215 |
| rs9605980 | 0.503 | 0.1 |
| rs878825 | 3.445 | 0.038 |
| rs6000820 | 1.231 | 0.532 |
| rs139954 | 3.024 | 0.212 |
| rs926299 | 0.178 | 0.741 |

**∑χ^2^ =** 353.603 (434 df), Global P-value (after 1000 simulations for each locus) = 0.9981

**Table S2**: Number statistics of prioritized SNPs for five genes

| **Gene Symbol** | **Total SNPs** | **SNPs failed in assay designing** | **SNPs failed in genotyping** | **Passed SNP** | **SNPs Out of HWE** | **SNPs before LD pruning** | **SNPs with MAF<0.1** | **SNPs with MAF>0.1** | **SNPs in LD (r^2^≥9)** | **SNPs included in analysis** |
| --- | --- | --- | --- | --- | --- | --- | --- | --- | --- | --- |
| ***RGS4*** | 26 | 0 | 5 | 21 | 1 | 20 | 5 | 15 | 5 | 10 |
| ***SLC6A3*** | 27 | 2 | 3 | 22 | 1 | 21 | 4 | 17 | 3 | 14 |
| ***PIP5K2A*** | 25 | 1 | 1 | 23 | 4 | 19 | 2 | 17 | 3 | 14 |
| ***BDNF*** | 19 | 0 | 1 | 18 | 0 | 18 | 2 | 16 | 6 | 10 |
| ***PI4KA*** | 20 | 1 | 2 | 17 | 2 | 15 | 4 | 11 | 6 | 5 |
| **Total** | **117** | **4** | **12** | **101** | **8** | **93** | **17** | **76** | **23** | **53** |

Footnote: SNPs, Single nucleotide polymorphism; HWE, Hardy-Weinberg equilibrium; LD, Linkage disequilibrium; MAF, Minor allele frequency; r^2^; correlation coefficient, measurement of LD

**Table S3: Details and allele frequencies of polymorphisms in schizophrenia patients (n=482) and controls (n=230)**

| **dbSNP id**  **(Built-136)** | **Chromosomal Position** | **Region (effect)** | **Function prediction^a^** | **Alleles^b^** | **MAF^c^ in patients** | **MAF^c^ in controls** |
| --- | --- | --- | --- | --- | --- | --- |
| **Regulator of G protein signaling 4 (*RGS4*) -1q23.3** | | | | | | |
| rs2842017 | 163026652 | 5' Near gene | Conserved, Chromatin_Structure|DNase-seq, Protein_Binding|ChIP-seq|JUN, JUND | C/T | 0.26 | 0.2 |
| rs2842026 | 163033089 | 5' Near gene | Conserved, Chromatin_Structure|DNase-seq, Motifs |Irf_disc5, Pax-5_disc3, TATA_disc7,Tel2, p300_disc5 | A/C | 0.27 | 0.28 |
| rs951439 | 163033691 | 5' Near gene | Conserved,TFBS, Motifs|PWM|Pbx3_disc2, SMAD4, ZBRK1, Chromatin_Structure| DNase-seq | T/C | 0.49 | 0.48 |
| rs6427711 | 163034416 | 5' Near gene | Motifs|BDP1_disc1, DP1_disc1, Cdx2_2, Hoxa9, Hoxb13, Hoxb9,Hoxd10, TATA_known5| | G/A | 0.45 | 0.45 |
| rs6678136 | 163037317 | 5' Near gene | Conserved,TFBS, Motifs|FAC1, Foxp3, Hoxa9, Hoxb9| | A/G | 0.49 | 0.49 |
| rs7515900 | 163038613 | 5' Near gene | Conserved,TFBS, Chromatin_Structure| FAIRE, DNase-seq, Motif|Nkx6-2, TATA_known3| | A/C | 0.48 | 0.49 |
| rs2661319 | 163039777 | Intron 1 | Conserved, Chromatin_Structure|DNase-seq, Protein_Binding|ChIP-seq|GATA1, GATA2 | A/G | 0.25 | 0.29 |
| rs2842030 | 163040495 | Intron 1 | Chromatin_Structure|DNase-seq | G/T | 0.29 | 0.33 |
| rs10799897 | 163043088 | Intron 3 | Conserved, Motifs |GATA,Hoxa7| | G/A | 0.29 | 0.29 |
| rs10759 | 163046351 | 3' UTR | Conserved, ESE, miRNA, Motifs|Esr1_2 ,Dax1, Motifs|PWM|ER, Chromatin_Structure|DNase-seq | C/A | 0.31 | 0.3 |
| **Solute Carrier Family 6 (Neurotransmitter Transporter, Dopamine), Member (*SLC6A3*)-5p15.3** | | | | | | |
| rs3756450 | 1448148 | 5' Near gene | Conserved,TFBS, Chromatin_Structure| FAIRE, DNase-seq, Protein_Binding| ChIP-seq|ETS1|Motifs|Cart1, Foxo_2, GATA_known6, GATA_known9, Nanog_disc2| | T/C | 0.35 | 0.3 |
| rs2550956 | 1447841 | 5' Near gene | TFBS, Chromatin_Structure|FAIRE, DNase-seq | C/T | 0.28 | 0.25 |
| rs2975226 | 1445616 | 5' Near gene | Conserved,TFBS, Motifs|PWM|AP-2_known3, E2F_disc3, Rad21_disc6, UF1H3BETA, Protein_Binding|ChIP-seq|EGR1, POLR2A | T/A | 0.45 | 0.46 |
| rs2975223 | 1443603 | Intron 1 | Conserved, Chromatin_Structure|FAIRE, DNase-seq| Motif|DBP, LBP-1_3, MAZR, MZF1::1-4_2, PU.1_disc3, SP1_disc3, SP1_known1, STAT_disc7, TATA_disc7, VDR_2, ZNF263_disc1, Znf143_disc3| | G/A | 0.5 | 0.49 |
| rs420422 | 1436408 | Intron 3 | Chromatin_Structure|FAIRE, DNase-seq | G/A | 0.42 | 0.41 |
| rs463379 | 1431164 | Intron 4 | Chromatin_Structure|FAIRE, DNase-seq | C/G | 0.45 | 0.49 |
| rs10052016 | 1428111 | Intron 4 | Conserved, Motifs|LXR_2, LXR_3, Nrf1_known2, RAR| | A/G | 0.16 | 0.13 |
| rs464049 | 1423905 | Intron 4 | Conserved, Motif |DMRT5, MIF-1, Mxi1_disc1, Myc_disc4, NF-I_2, Pax-5_disc1, RFX5_known2, SREBP_disc1,| | C/T | 0.34 | 0.35 |
| rs11564758 | 1420588 | Intron 6 | Conserved, Motifs|PWM|Six4, Nr2f2, SIX5_known1, Tgif1_1| Chromatin_Structure| DNase-seq | G/C | 0.21 | 0.2 |
| rs37022 | 1415629 | Intron 7 | Conserved, Motifs|GR_disc1, GR_known3, GR_known4, GR_known7, Nanog_disc2, Sox_4, GR, Sox2, Gm397, Chromatin_Structure|DNase-seq | T/A | 0.31 | 0.34 |
| rs27048 | 1412645 | Intron 8 | Conserved, Motifs |Smad_2|, Chromatin_Structure|DNase-seq | C/T | 0.3 | 0.27 |
| rs6347 | 1411412 | Exon 9 (Ser405Ser) | Conserved,ESE, Motif| GR_disc6, NRSF_disc8, Zfx |FAIRE, Chromatin_Structure| DNase-seq | A/G | 0.15 | 0.12 |
| rs40184 | 1395077 | Intron 14 | Chromatin_Structure|FAIRE, DNase-seq| Motifs|BATF_disc2, Pou5f1_known2| | G/A | 0.38 | 0.36 |
| rs27072 | 1394522 | 3' UTR | Conserved, miRNA, |HAX1|eQTL, Motifs|PWM|BHLHE40_disc2, CHD2_disc3, GR_disc6, HNF4_known4, NRSF_disc5, NRSF_known1, Znf143_disc4, p300_disc9, HNF4| COUPTF, Chromatin_Structure |FAIRE, DNase-seq | C/T | 0.23 | 0.25 |
| **Phosphatidylinositol-5-Phosphate 4-Kinase, Type II, Alpha (*PIP5K2A*)-10p12.2** | | | | | | |
| rs1417374 | 23128475 | 5' Near gene | Conserved, Motifs|NF-I| | C/T | 0.34 | 0.36 |
| rs946961 | 23094828 | 5' Near gene | Conserved | C/G | 0.38 | 0.41 |
| rs997650 | 22986018 | Intron 1 | Motifs|PWM|HeliosA|EWSR1-FLI1|Ik-2_3| | T/C | 0.13 | 0.19 |
| rs1171506 | 22951867 | Intron 1 | Conserved, Chromatin_Structure|FAIRE, DNase-seq, Protein_Binding|ChIP-seq|GATA1,|TAL1, GATA2| Motif|Egr-1_known1, Egr-1_known2| | G/A | 0.21 | 0.22 |
| rs2559524 | 22892152 | Intron 3 | Conserved, Motifs|AP-1_disc7, Pax-2_3, Pax-3_1, Pax-5_disc2| | G/A | 0.15 | 0.19 |
| rs1409396 | 22879032 | Intron 4 | Conserved, Motifs|PWM|CLOCK:BMAL, BHLHE40_known2, Myc_known3, NF-E2_disc3| Chromatin_Structure|FAIRE, Chromatin_Structure| DNase-seq | T/C | 0.38 | 0.43 |
| rs11013052 | 22862487 | Intron 4 | Conserved, Motifs|PWM|CIZ, HDAC2_disc5, Irf_known3, PTF1-beta,| Chromatin_Structure| FAIRE | C/A | 0.2 | 0.2 |
| rs2296624 | 22856946 | Intron 5 | Conserved | G/A | 0.19 | 0.2 |
| rs7094131 | 22847439 | Intron 6 | Conserved, TFBS, Motifs|PWM|Tcf7l2, DMRT3, Tcf3, Lef1, Tcf7, DMRT4, Pou2f1_4, DMRT2, DMRT3, DMRT4, Foxp1, Nkx6-1_1, Pbx-1_4, Pou2f2_known10, Pou2f2_known2, Pou5f1_known1, SIX5_known2, TCF4_known1 | T/C | 0.21 | 0.21 |
| rs10828317 | 22839628 | Exon 7 (Asn251Ser) | Conserved, Omega, Oreganno, ESE, abolished, Benign, |eQTL, Motifs|PWM |Foxj1, FAC1, Maf_known4, Zfp105| , Chromatin_Structure| DNase-seq | T/C | 0.19 | 0.17 |
| rs10828316 | 22838389 | Intron 7 | Conserved, Motifs|PWM|Hbp1, Irf_known3, Pax-8_1| | A/C | 0.19 | 0.19 |
| rs746203 | 22830541 | Intron 8 | eQTL, Chromatin_Structure |FAIRE, DNase-seq | A/G | 0.26 | 0.26 |
| rs1053454 | 22826093 | 3' UTR | Conserved, miRNA binding site|Motifs|ERalpha-a_disc4, Pbx3_disc2| | G/T | 0.22 | 0.2 |
| rs45627240 | 22825887 | 3' UTR | Conserved, Motifs|PWM|MAZR, Sp1_1, SP1, RREB1, Zfp740,Zfp281, WT1,RREB-1, WT1, Zfp281, MAZR, ZNF219, Sp1, CHD2_disc3, Egr-1_known2, Foxp1, Glis2, MAZR, RREB-1_1, RREB-1_2, SP1_known1, SP1_known4, STAT_disc7, UF1H3BETA, WT1, ZBTB7A_known2, ZNF219, Zfp281, Zfp740 | Del/C | 0.23 | 0.29 |
| **Brain-Derived Neurotrophic Factor (*BDNF*)- 11p13** | | | | | | |
| rs4923463 | 27672500 | 5' Near gene | Conserved, Protein_Binding|ChIP-seq| RFX3 | A/G | 0.3 | 0.29 |
| rs1491851 | 27752763 | 5' Near gene | might influence the density of two proteins 5-HTT and 5-HT1A expressed by brain serotonergic neurons | T/C | 0.38 | 0.39 |
| rs56164415 | 27721735 | 5'UTR | Chromatin_Structure|FAIRE, DNase-seq, Motifs|BCL_disc8, Myc_known6, Znf143_disc3| | G/A | 0.22 | 0.21 |
| rs11030121 | 27736207 | Intron 1 | Conserved, Motifs|PWM|Sox13, HNF1_1, HNF1_7| | C/T | 0.39 | 0.36 |
| rs7127507 | 27714884 | Intron 1 | Conserved, Motifs |CTCF_disc10, Foxa_disc3, GR_disc5, Maf_known4| | T/C | 0.39 | 0.38 |
| rs7103411 | 27700125 | Intron 1 | Conserved, Chromatin_Structure|DNase-seq, motifs |DMRT5| | T/C | 0.34 | 0.34 |
| rs11030101 | 27680744 | Intron 1 | Conserved, Motifs |HDAC2_disc6| | A/T | 0.26 | 0.3 |
| rs6265 | 27679916 | Exon 1 (Val66Met) | affects intracellular trafficking and packaging of proBDNF molecules and the methionine variant shows a lower depolarization-induced secretion of the protein in cultured hippocampal neurons; Conserved, Omega,Benign, Motifs|PWM| MAX, CLOCK:BMAL, BHLHE40_known2, Myc_disc7, Myc_known3, Myc_known5, Myc_known7, Myc_known9, SREBP_known4| Chromatin_Structure|FAIRE | C/T | 0.3 | 0.26 |
| rs7124442 | 27677041 | 3' UTR | Increases BDNF plasma levels, Conserved, miRNA site | T/C | 0.4 | 0.39 |
| rs1519480 | 27675712 | 3' UTR | Conserved, Chromatin_Structure|FAIRE, DNase-seq, Protein_Binding|ChIP-seq|YY1, GATA2, Motifs|Gfi1_1, Hoxb8, SP1_disc2| | T/C | 0.37 | 0.37 |
| **Phosphatidylinositol 4-Kinase, Catalytic, Alpha (*PI4KA*)- 22q11.21** | | | | | | |
| rs165729 | 21188732 | Intron 3 | Conserved, Chromatin_Structure|FAIRE, DNase-seq, Motifs |Myf_4| | C/A | 0.38 | 0.41 |
| rs165854 | 21167787 | Exon 8 (Cys288Cys) | Conserved, ESE, Motifs|TCF12_disc5, Znf143_known1| | G/A | 0.34 | 0.35 |
| rs165811 | 21163327 | Intron 9 | Motifs|Footprinting|SEF-1, |PWM|SEF-1, BAF155_disc2, Ets_known3, Evi-1_5, Foxp3, GATA_disc3, Myf_1, PEBP, SEF-1, SIX5_disc1, Znf143_disc2, Chromatin_Structure| DNase-seq, Protein_Binding|ChIP-seq| EBF1 | C/T | 0.28 | 0.29 |
| rs165793 | 21147293 | Intron 19 | Chromatin_Structure|DNase-seq|Motifs|Pax-4_1| | G/A | 0.28 | 0.26 |
| rs2072513 | 21099297 | Intron 29 | eQTL|Motifs|Pax-2_1, ERalpha-a_disc1| | T/C | 0.31 | 0.33 |

Footnote: ^a^From RegulomeDB and HaploReg; ^b^Major allele/minor allele; ^c^Minor allele frequency

**Table S4: Global frequency and world-wide frequency of major allele six drug response associated polymorphisms of *PIP4K2A* typed in 1000 Genomes Project**

| **Populations** | **N** | **rs10828317 (T/C)** | | **rs11013052 (C/A)** | | **rs1409396 (A/G)** | | **rs2296624 (C/T)** | | **rs7094131 (C/T)** | | **rs746203 (C/T)** | |
| --- | --- | --- | --- | --- | --- | --- | --- | --- | --- | --- | --- | --- | --- |
| **Global** | 1093 | T | 0.74 | C | 0.78 | A | 0.70 | C | 0.69 | C | 0.51 | T | 0.56 |
| **AFRICA** | 246 | T | 0.92 | C | 0.72 | A | 0.62 | C | 0.71 | C | 0.79 | C | 0.67 |
| **EUROPE** | 380 | T | 0.67 | C | 0.66 | A | 0.54 | C | 0.65 | T | 0.59 | T | 0.60 |
| **EAST ASIA** | 286 | T | 0.59 | C | 0.95 | A | 0.93 | C | 0.61 | T | 0.51 | T | 0.59 |
| **AMERICA** | 181 | T | 0.88 | C | 0.85 | A | 0.76 | C | 0.85 | T | 0.64 | T | 0.74 |
| ASW | 61 | T | 0.86 | C | 0.72 | A | 0.60 | C | 0.73 | C | 0.74 | C | 0.62 |
| LWK | 97 | T | 0.92 | C | 0.68 | A | 0.56 | C | 0.66 | C | 0.84 | C | 0.69 |
| YRI | 88 | T | 0.95 | C | 0.77 | A | 0.71 | C | 0.74 | C | 0.77 | C | 0.68 |
| CEU | 87 | T | 0.60 | C | 0.62 | A | 0.52 | C | 0.58 | T | 0.53 | T | 0.52 |
| FIN | 93 | T | 0.63 | C | 0.65 | A | 0.55 | C | 0.63 | T | 0.54 | T | 0.55 |
| GBR | 88 | T | 0.63 | C | 0.63 | A | 0.52 | C | 0.63 | T | 0.61 | T | 0.61 |
| IBS | 14 | T | 0.75 | C | 0.71 | A | 0.61 | C | 0.75 | T | 0.64 | T | 0.64 |
| TSI | 98 | T | 0.79 | C | 0.74 | A | 0.56 | C | 0.75 | T | 0.67 | T | 0.70 |
| CHB | 97 | T | 0.59 | C | 0.94 | A | 0.92 | C | 0.62 | T | 0.52 | T | 0.59 |
| CHS | 100 | T | 0.57 | C | 0.94 | A | 0.91 | C | 0.57 | T | 0.53 | T | 0.55 |
| JPT | 89 | T | 0.63 | C | 0.98 | A | 0.96 | C | 0.64 | C | 0.53 | T | 0.63 |
| CLM | 60 | T | 0.88 | C | 0.82 | A | 0.71 | C | 0.86 | T | 0.62 | T | 0.72 |
| MXL | 66 | T | 0.90 | C | 0.92 | A | 0.85 | C | 0.88 | T | 0.65 | T | 0.81 |
| PUR | 55 | T | 0.86 | C | 0.78 | A | 0.71 | C | 0.82 | T | 0.66 | T | 0.69 |
| **South Indian patients** | 482 | T | 0.81 | C | 0.80 | A | 0.62 | C | 0.81 | T | 0.79 | T | 0.74 |
| **South Indian controls** | 230 | T | 0.83 | C | 0.80 | A | 0.57 | C | 0.80 | T | 0.79 | T | 0.74 |

Footnote: N, number of individuals;

Major allele and major allele frequencies were provided in this table.
